# LncRNA CARMN overexpression promotes prognosis and chemosensitivity of triple negative breast cancer via acting as miR143-3p host gene and inhibiting DNA replication

**DOI:** 10.1186/s13046-021-02015-4

**Published:** 2021-06-23

**Authors:** Xiaonan Sheng, Huijuan Dai, Yueyao Du, Jing Peng, Rui Sha, Fan Yang, Liheng Zhou, Yanping Lin, Shuguang Xu, Yifan Wu, Wenjin Yin, Jinsong Lu

**Affiliations:** Department of Breast Surgery, Renji Hospital, School of Medicine, Shanghai Jiao Tong University, Shanghai, P.R. China

**Keywords:** lncRNA, miRNA, Triple negative breast cancer, Neoadjuvant chemotherapy, Predictive and prognostic biomarker, Host gene

## Abstract

**Background:**

Triple negative breast cancer (TNBC) is a subtype of breast cancer with poor prognosis and lack of effective treatment target. Here we screened differentially expressed lncRNAs through bioinformatics analysis and identified CARMN as a downregulated lncRNA which is lowest expressed in TNBC. We aimed to identify the potential role and molecular mechanisms of CARMN in TNBC.

**Methods:**

Predictive value of CARMN was explored in breast cancer cohorts. TNBC cell lines with CARMN overexpression or CARMN silence and were used for in vitro and in vivo experiments. RNA-seq of CARMN overexpressed cells was performed for exploring downstream of CARMN.

**Results:**

CARMN is downregulated at different phase of malignant transformation of breast tissue. CARMN can predict both better prognosis and higher response rate of cisplatin-based neoadjuvant chemotherapy in breast cancer. A nomogram is built to predict cisplatin-based chemotherapy response in breast cancer. Through in vitro and in vivo studies, we confirmed CARMN can also inhibit tumorigenesis and enhance sensitivity to cisplatin in TNBC cells. RNA-seq and further experiments revealed CARMN can inhibit DNA replication. MCM5, an important DNA replication initiation factor, is the most downregulated gene in DNA replication pathway following CARMN overexpression. We confirmed CARMN can produce miR143-3p from its exon5 which is DROSHA and DICER dependent, resulting binding and decrease of MCM5. Moreover, suppressing miR143-3p can weaken function of CARMN in suppressing tumorigenesis and promoting chemosensitivity.

**Conclusions:**

Our results indicated lncRNA CARMN is a predictive biomarker of better prognosis and enhanced cisplatin sensitivity in TNBC. CARMN is the host gene of miR143-3p which downregulates MCM5, causing inhibited DNA replication.

**Supplementary Information:**

The online version contains supplementary material available at 10.1186/s13046-021-02015-4.

## Background

Triple negative breast cancer (TNBC) with estrogen receptor (ER), progesterone receptor (PR) and human epidermal growth factor receptor 2 (HER2) all negative accounts for 15% of all breast cancer. Compared with other subtypes of breast cancer, TNBC is more likely to appear in young patients, with the worst prognosis due to its high capability of invasion, heterogeneous behavior and lack of effective treatment target [1]. To date, chemotherapy is still the main treatment for TNBC patients. However, clinical benefit of chemotherapy for TNBC is often blocked by the chemoresistance [2]. Thus, increasing sensitivity of existed chemotherapy regimens and exploring novel therapeutic targets to improve the overall prognosis for TNBC are of urgent.

Growing evidences showed long non-coding RNAs (lncRNAs) act as critical factors in tumorigenesis and tumor progression as well as therapeutic sensitivity [3, 4]. Although with no or low capability to translate into protein, lncRNAs play a pivotal role in many biological processes and diseases development [5]. However, molecular mechanism for lncRNAs regulating TNBC progression and chemotherapy resistance are still not fully understood. LncRNA Cardiac Mesoderm Enhancer-associated Noncoding RNA (CARMN) is a lncRNA extremely downregulated in TNBC tissues. CARMN located at chromosome 5q32 and was first reported in 2015 [6] as a cell identity related gene expression, regulating differentiation of human cardiac precursor cell. Till now, CARMN’s role in breast cancer has never been reported.

Here, we initially demonstrated that CARMN was decreased along with breast tissue malignant transformation whose overexpression was linked to better prognosis and better response of cisplatin-based neoadjuvant chemotherapy in breast cancer. Further experiments demonstrated CARMN synergistically inhibit cancer development and increase cisplatin sensitivity in TNBC by regulating essential pathways in tumorigenesis and cisplatin related processes, especially inhibiting DNA replication. Mechanistically, CARMN acts as host gene of miR143-3p which targets an essential DNA replication license gene MCM5. Restoration of CARMN is a potential target for breast malignant transformation, cancer development and enhancing cisplatin sensitivity.

## Methods

### Patients

Patients whose tumor tissues were used for evaluating the role of CARMN in predicting response to neoadjuvant chemotherapy all participated in a randomized, open-label, prospective phase III clinical trial of Renji hospital, in which patients received weekly paclitaxel and cisplatin as neoadjuvant chemotherapy for operable local-advanced breast cancer. This study was registered with ClinicalTrials.gov. as NCT02221999 (date of registration: August 21, 2014, https://clinicaltrials.gov/ct2/show/NCT02221999?term=NCT02221999&draw=2&rank=1). Inclusion and eligible criteria mainly include: 18 to 70 years old; with large operable breast cancer (T_2-4_N_0-3_M_0_); without prior systemic therapy for breast cancer or tolerate with chemotherapy. All the patients hospitalized and were diagnosed breast cancer with Elite biopsy system as previously described [7].

Paired breast cancer and normal breast tissues were also obtained from patients who underwent mastectomy or modified radical mastectomy after diagnosed as breast cancer with biopsy before surgery in Renji Hospital. This study was approved by the Ethics Committee of Renji Hospital (No.2017–088) following the principles of the Helsinki Declaration. All participants provided written informed consent to take part in the study.

### Cell culture

Breast cancer cell lines and normal breast cell MCF10A were obtained from Renji Hospital, Shanghai Jiao Tong University School of Medicine. MCF10A was cultured with DMEM medium (Gibco, USA) with 5% horse serum (Gibco, USA), hydrocortisone (0.5 lg/ml), insulin (10 lg/ ml), EGF (20 ng/ml), cholera toxin (100 ng/ml) and 1% penicillin-streptomycin. Other cell lines were cultured with DMEM medium (Gibco, USA) with 10% Fetal Bovine Serum (Gibco, USA) and 1% penicillin-streptomycin in 37 °C with 5% CO2.

### RNA isolation and qRT-PCR

Total RNA of tissues or cells was extracted by TRIzol (Invitrogen, USA). RNA of nuclear and cytoplasm were separated with separation kit purchased from Norgenbiotek (Canada, Lot 594,895). Isolated RNA was reverse-transcribed into cDNA with Reverse Transcription Kit (Takara, Japan). Reverse-transcription of miRNA was done with miRcute plus miR first-strand cDNA synthesis kit (Tiangen, China). QPCR was done with SYBR Premix Ex TaqII (Takara, Japan) and miRcute plus miR qPCR detection kit (Tiangen, China). RT-qPCR result was detected with light cycler instrument (LightCycler 480 II, Roche, Germany). The expression of lncRNA and protein coding RNA was normalized by β-actin and miRNA expression was normalized by U6. The primers of detected RNAs are listed in Supplementary Table 1. Expression of detected RNAs was calculated by 2^-ΔΔCT^.

### Cell transfection with lentivirus, lncRNA smart silencer, miRNA inhibitor, miRNA mimics siRNA and plasmid

Cells were transfected with lentivirus vectors overexpressing CARMN or control ones (purchased from GenePharma Shanghai, China) for stably overexpressing CARMN. CARMN smart silencer [8] was bought from Ribobio (Guangzhou, China).

MiR143-3p inhibitor and inhibitor control (GenePharma, Shanghai, China), miR143-3p mimics (purchased from Genomeditech, China), siRNA of DROSHA/DICER1/MCM5 (purchased from Genomeditech, China), plasmid overexpressing exon5 of CARMN (purchased from Genechem, Shanghai, China), whose sequence are all listed in Supplementary Table 2, were transfected with Polyplus Transfection jetPRIME (France). After 48 h, the cells were collected for detecting efficiency and further use.

### Cell proliferation and migration assay

Cell proliferation was detected at 0 h, 24 h, 48 h and 72 h was detected with Cell Counting Kit-8 (CCK-8, Dojindo, Japan). For colony formation assay, cells were seeded in 6-well plate and were fixed, stained and observed under microscope after 14 days. For migration assay, cells were seeded in 24-well transwell plate upper chamber. Cells were cultured in serum-free medium in the upper chamber and medium with serum in the lower chamber. After 24 h, the cells at bottom of the filters were fixed and stained for obtaining and counting.

### Drug sensitivity assay

Cells were treated with different concentration of cisplatin (CSNpharm, Chicago, USA): 100, 50, 25, 12.5, 6.25, 3.13, 1.56, 0.78, 0.39, 0.20, 0.10 and 0 μg/ml). Cell activity was detected by CCK8 assay after 24 h.

### EdU cell proliferation assay

Cells were treated with 5 μg/ml cisplatin or blank for 24 h. Cells were stained with EdU with EdU Cell Proliferation Kit with Alexa Fluor 488 (BeyoClick, China) following manufacture’s protocol. Hoechst was stained as well for identifying cell nucleus.

### Western blot and antibodies

Primary antibodies of MCM5 (11703–1-AP, anti-rabbit 1500) and ACTIN (ab49900, HRP, 1:1000) were purchased from Proteintech (Chicago, USA) and Abcam (Cambridge, UK) respectively. Primary antibodies of DROSHA (AF6732, 1:500) and DICER1 (AF6702, 1:500) were purchased from Beyotime (Shanghai, China). Secondary antibody (A0208, 1:2000) was bought from Beyotime (Shanghai, China). Enhanced chemiluminescence (ECL) was performed with Immobilon Western Chemiluminescent HRP Substrate (Millipore, Billerica, USA) and Chemidoc Touching Imaging System (Biorad, California, USA) was used for observing results.

### Immunofluorescence

The cells were stained with DAPI stain and β-tubulin antibody (Servicebio, Hubei, Wuhan). Immunofluorenscence antibody of MCM5 (11703–1-AP, anti-rabbit 1:100) was purchased from Proteintech (Chicago, USA). Secondary antibody with Alexa Fluor 488(A0423, 1:500) was purchased from Beyotime (Shanghai, China).

### Cell cycle and apoptosis assay

For cell cycle assay, cells were collected and stained with propidium iodide (PI) using Cell Cycle Assay Kit - PI/RNase Staining (Dojindo, Japan) following manufacture’s protocol. For cell apoptosis assay, cells were treated with cDDP (10 μg/ml) or blank control for 24 h. Later, cells were labeled with Annexin-V FITC and PI using Apoptosis Detection Kit (Dojindo, Japan). LSRFortessa (BD) was then used for flow cytometry analysis.

### RNA transcriptome sequencing

Sequencing libraries generation was performed using NEBNextR UltraTM Directional RNA Library Prep Kit for IlluminaR (NEB, USA) following manufacturer’s protocol. Illumina Hiseq Xten platform was used for library preparations sequence. Gene expression levels were calculated by fragments per kilobase of transcript per million fragments mapped (FPKM). Differential expression analysis of two different CARMN expression condition groups was performed with R package “DESeq” under threshold of adjusted *P*-value < 0.05 and absolute value of fold change> 1.5.

### Luciferase reporter assay

Binding site of miR143-3p and MCM5 was predicted with STARBASE [9] (http://starbase.sysu.edu.cn/index.php). Luciferase reporter vectors (pMIR-CMV Vector, Promega, Madison, WI, USA) with wild-type MCM5 or mutant MCM5 in predicted binding site were constructed. MDA-MB-231 cells were co-transfected with luciferase reporter vectors and miR143-3p or negative control mimics. Reporter luciferase activity was detected after 48 h by Dual-Luciferase Reporter Assay System (Promega) with Renilla luciferase activity for normalization.

### Experiments in vivo

Female BALB/c nude mice (SLAC, Shanghai, China) were used for breast cancer xenograft model. CARMN overexpressed or control MDA-MB-468 cells was injected into subcutaneous flanks of 5-week-old nude mice. All mice were sacrificed 6 weeks after injection and xenografts were taken out for weighting. Experiments in vivo were performed following protocol approved by Shanghai Jiao Tong University Institutional Animal Care and Use Committee and Renji Hospital Animal Care guidelines. To minimize animal suffering, all efforts were made.

### TUNEL assay

TUNEL assay of xenografts was implied with TUNEL kit from Roche (Basel, Switzerland) in xenografts following manufacture’s protocol.

### Bioinformatic analysis

TCGA and GEO datasets were analyzed by R packages “TCGAbiolinks” and “limma”, also online tool GEPIA (http://gepia.cancer-pku.cn/index.html) [10]. The survival of breast cancer cohort was analyzed with K-M plotter (http://kmplot.com/analysis/index.php?p=background) [11]. KEGG and GO pathway enrichment was carried out with R package “clusterProfiler”, “DOSE” and “enrichplot” [12, 13]. GSEA pathway enrichment was carried out with software GSEA (version 4.0.1) [14]. Genome information of CARMN and miRNA was obtained from ENSEMBL (http://uswest.ensembl.org), NCBI gene (https://www.ncbi.nlm.nih.gov). and miRbase (http://www.mirbase.org).

### Statistical analysis

Statistical analysis and figures were conducted with R (3.4.3) and Graphpad Prism (8.1.1). Two-side *p* value < 0.05 was considered as statistically significant.

More details of materials and methods are described in Supplementary Materials and Methods.

## Results

### LncRNA CARMN is a biomarker for prognosis and malignant transformation in breast cancer

**Fig. 1:**
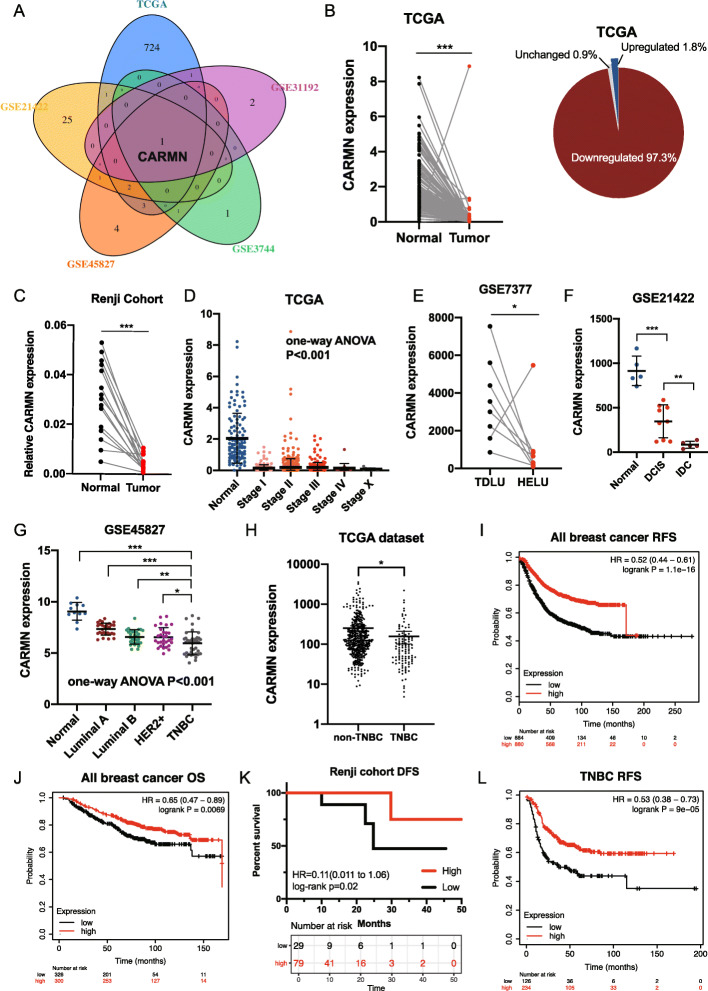
LncRNA CARMN decreases with malignant change of breast tissue and predicts prognosis in breast cancer. **A** Venn diagram for screening lncRNAs differently expressed in breast cancer from normal breast tissue in TCGA database, GSE45827, GSE31192, GSE3744 and GSE21422, respectively. LncRNA CARMN is decreased in tumor tissue in all 5 databases. **B** CARMN expression in paired breast cancer and normal breast tissue in TCGA database and distribution of changing tendency. **C** Expression of CARMN detected by RT-qPCR in paired breast cancer and normal breast tissue from Renji cohort. **D** Expression of CARMN in different clinical stages of breast cancer in TCGA. **E** CARMN expression in paired hyperplastic enlarged lobular units (HELU) and terminal duct lobular units (TDLU) in GSE7377. **F** CARMN expression in normal breast tissue, ductal carcinoma in situ (DCIS) and invasive ductal carcinoma (IDC) in GSE21422. **G** Expression of CARMN in different subtypes of breast cancer according to GSE45827. **H** Comparison of CARMN expression in TNBC and non-TNBC according to TCGA dataset. **I** Relapse-free survival (RFS) of all breast cancer patients with different expression of CARMN according to Kaplan-Meier Plotter. **J** Overall survival (OS) of all breast cancer patients with different CARMN expression according to Kaplan-Meier Plotter. **K** Disease-free survival (DFS) of patients with different expression of CARMN who received neoadjuvant chemotherapy in Renji cohort. **L** Recurrence-free survival (RFS) of TNBC patients with different expression of CARMN according to Kaplan-Meier Plotter. T: tumor; N: normal; ER: estrogen receptor; HER2: human epidermal growth factor receptor 2; TNBC: triple negative breast cancer; HELU: hyperplastic enlarged lobular units; TDLU: terminal duct lobular units; RFS:Relapse-free survival; OS: Overall survival; DFS, Disease-free survival. Error bars represent means ± SD, *P < 0.05, **P < 0.01, ***P < 0.001

Differentially expressed lncRNAs between breast cancer and normal breast tissues were screened from TCGA dataset, GSE45827, GSE31192, GSE3744 and GSE21422 (FDR < 0.05, |log_2_FC| > 2). Only one lncRNA CARMN showed differently expressed in all 5 datasets with the same tendency (Fig. 1A).

Decrease of CARMN in breast cancers samples comparing with paired normal tissues was confirmed in two cohorts (Fig. 1B, C, Figure S1A) with outstanding capability in discriminating tumor from normal tissue evaluated by ROC curve (Figure S1B). In TCGA dataset, CARMN was downregulated in each stage of breast cancer comparing with normal samples (Fig. 1D). More evidences confirmed that CARMN is closely correlated with different phase of malignant transformation. In GSE7377, CARMN shows lower expression in premalignant hyperplastic enlarged lobular units (HELU) than in paired normal terminal duct lobular units (TDLU) (Fig. 1D, Figure S1C). In GSE21422, CARMN in ductal carcinoma in situ (DCIS) was lower than that in normal breast tissue and was obviously higher than that in invasive ductal carcinoma (IDC) at the same time (Fig. 1E). Besides breast cancer, CARMN is also significantly downregulated in many other cancers (Figure S1D).

CARMN was differently expressed in different subtypes of breast cancer (Fig. 1G), in which TNBC has the lowest expression of CARMN and luminal A breast cancer has the highest expression of CARMN. The low expression of CARMN in TNBC was also validated in TCGA dataset (Fig. 1H).

High expression of CARMN was also related with better prognosis in breast cancer patients, especially in patients with TNBC. Upregulation of CARMN is a strong predictor of longer relapse free survival and overall survival (Fig. 1I, J) in all breast cancer cohort and local advanced breast cancer cohort (Fig. 1 K). In TNBC cohort, CARMN also indicated longer relapse free survival (Fig. 1 L). However, survival benefit in ER+ or HER2+ breast cancer didn’t have statistical significance (Figure S1E, S1F).

### CARMN is a biomarker for high chemotherapy sensitivity in breast cancer

**Fig. 2:**
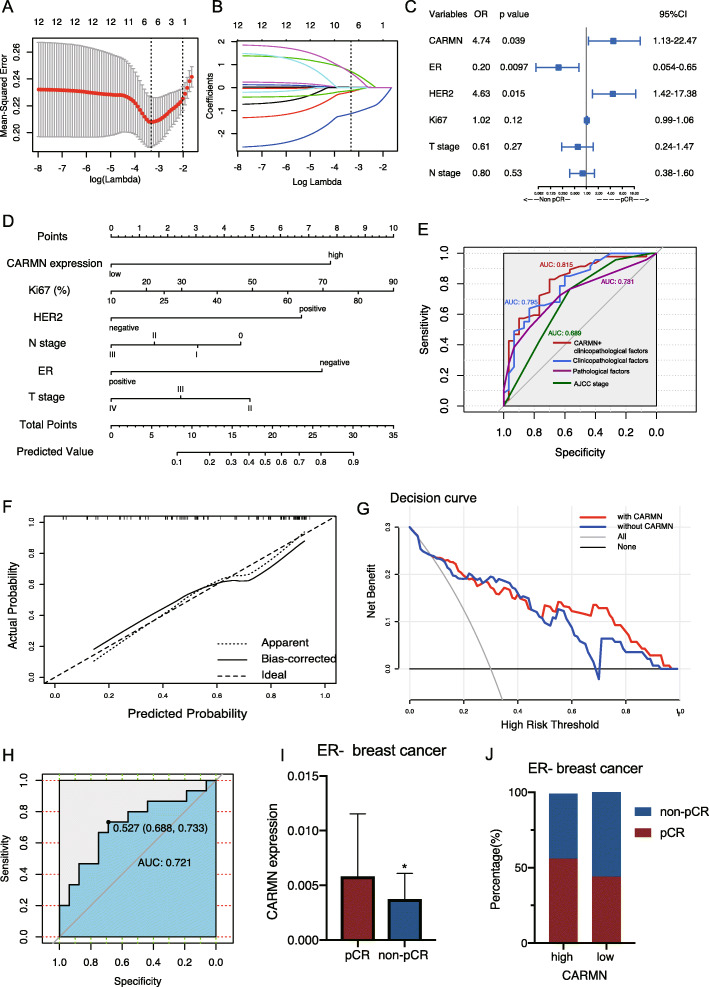
Nomogram established based on CARMN’s relationship with better cisplatin-based neoadjuvant chemotherapy response in breast cancer. **A**, **B** Texture feature selection for predicting response of neoadjuvant chemotherapy using lasso logistics regression. **A** Using minimum criteria of 10-fold cross-validation to select parameter lambda(λ) of LASSO model, and selected minimum λ turns out to be 0.0361. The upper x axis shows number of variants response to different λ values. The left dot line shows the minimum of λ and the right one shows λmin -SD. **B** LASSO coefficient profile plot of 12 texture features according to λ value selected in (**A**) which is also drawn as vertical line. Six optimal features are finally selected by lasso coefficient. **C** Forest plot illustrating factors selected by LASSO regression in predicting neoadjuvant chemotherapy response using multivariate logistics regression. **D** A nomogram is built for evaluating cisplatin-based neoadjuvant chemotherapy pCR rate based on multivariate logistics regression. **E** ROC curve of nomogram and other models for predicting cisplatin-based NAC response. **F** Calibration curve of the prediction model. X axis means the predicted probability of pCR. Y axis means the actual probability of pCR. The line between opposite angles means the ideal line. The other two lines means the actual prediction performance of the nomogram. **G** Decision curve of multivariate logistics model. The red line represents the multivariate logistics model including CARMN and clinicopathological factors, the blue one represents model with only clinicopathological factors. **H** ROC curve of nomogram in a validation set. **I** CARMN expression in ER- breast cancer with different neoadjuvant chemotherapy response. **H** Distribution of CARMN expression level in ER- breast cancer with different neoadjuvant chemotherapy response. ER: estrogen receptor; HER2: human epidermal growth factor receptor 2; TNBC: triple negative breast cancer; NAC: neoadjuvant chemotherapy; pCR: pathological complete response. Error bars represent means ± SD, *P < 0.05

In patients who received weekly cisplatin and paclitaxel as neoadjuvant chemotherapy (NAC) (Table 1), valuable factors in predicting NAC response including CARMN and other clinicopathological factors were selected with LASSO regression (Fig. 2A, B). CARMN was significantly related with NAC pathological completer response (pCR) in the multivariate logistics regression model combined with selected variables (Fig. 2C, Table 2). Then, we established nomogram for predicting possibility of NAC pCR with selected factors (Fig. 2D). With practical nomogram combing CARMN and clinicopathological factors, pCR rate could be predicted through total point of each single patient. ROC curve compared performance of different predicting models (Fig. 2E), showing model combining CARMN and clinicopathological factors has higher efficiency in pCR prediction. Calibration curve also indicated high efficiency of the model (Fig. 2F). According to decision curve (Fig. 2G), the model including CARMN and clinicopathological factors showed more clinical benefit than the model without CARMN. In another validation set, the prediction model exhibited sufficient AUC of ROC curve (Fig. 2H). Moreover, we also identified a significant upregulation of CARMN in ER- breast cancer with pCR (Fig. 2I, J).

**Table 1:** Baseline of training set and validation set

Variables	Training set (*n* = 77)	Validation set (*n* = 31)	*P*
Age	52.12 ± 9.97	51.03 ± 9.70	0.607
Menstruation			
Premenopause	34 (44%)	16 (52%)	0.572
Menopause	43 (56%)	15 (48%)	
T stage			
II	15 (19%)	3 (10%)	0.458
III	41 (53%)	18 (58%)	
IV	21 (27%)	10 (32%)	
N stage			
0	11 (14%)	2 (6%)	0.322
I	54 (70%)	20 (65%)	
II	3 (4%)	3 (10%)	
III	9 (12%)	6 (19%)	
BMI	23.96 ± 3.40	22.64 ± 2.49	0.052
ER			
≥10%	40 (52%)	15 (48%)	0.738
< 10%	37 (48%)	16 (52%)	
PR			
≥10%	39 (51%)	20 (65%)	0.238
< 10%	36 (47%)	11 (35%)	
HER2			
Positive	34 (44%)	11 (35%)	0.408
Negative	43 (56%)	20 (65%)	
Ki67 (%)	43.64 ± 20.69	39.84 ± 19.39	0.382
GNRHa			
Yes	20 (26%)	9 (29%)	0.812
No	57 (74%)	22 (71%)	
CARMN expression			
High	61 (79%)	21 (68%)	0.207
Low	16 (21%)	10 (32%)	
NAC Response			
Complete response	47 (61%)	15 (48%)	0.284
Partial response	30 (39%)	16 (52%)	

*BMI* body mass index, *ER* estrogen receptor, *PR* progesterone receptor, *HER2* human epidermal growth factor receptor 2, *GNRHa* Gonadotropin-releasing hormone analogue, *NAC* neoadjuvant chemotherapy

**Table 2:** Logistics regression of risk factors in predicting paclitaxel plus cisplatin neoadjuvant chemotherapy

	Univariate logistic regression		Multivariate logistic regression	
	OR(95%CI)	*P*	OR(95%CI)	*P*
CARMN	1.77 (0.58–5.47)	0.312	4.74 (1.13–22.47)	0.039
ER	0.17 (0.058–0.47)	< 0.001	0.20 (0.054–0.65)	0.0097
HER2	2.65 (1.03–7.23)	0.049	4.63 (1.42–17.38)	0.015
Ki67	1.03 (1.01–1.06)	0.014	1.02 (0.99–1.06)	0.12
T stage	0.57 (0.27–1.13)	0.11	0.61 (0.24–1.47)	0.27
N stage	0.64 (0.35–1.15)	0.14	0.80 (0.38–1.60)	0.53

*ER* estrogen receptor, *PR* progesterone receptor, *HER2* human epidermal growth factor receptor 2

### CARMN is a suppressor of TNBC and enhances cisplatin sensitivity in vivo and in vitro

**Fig. 3:**
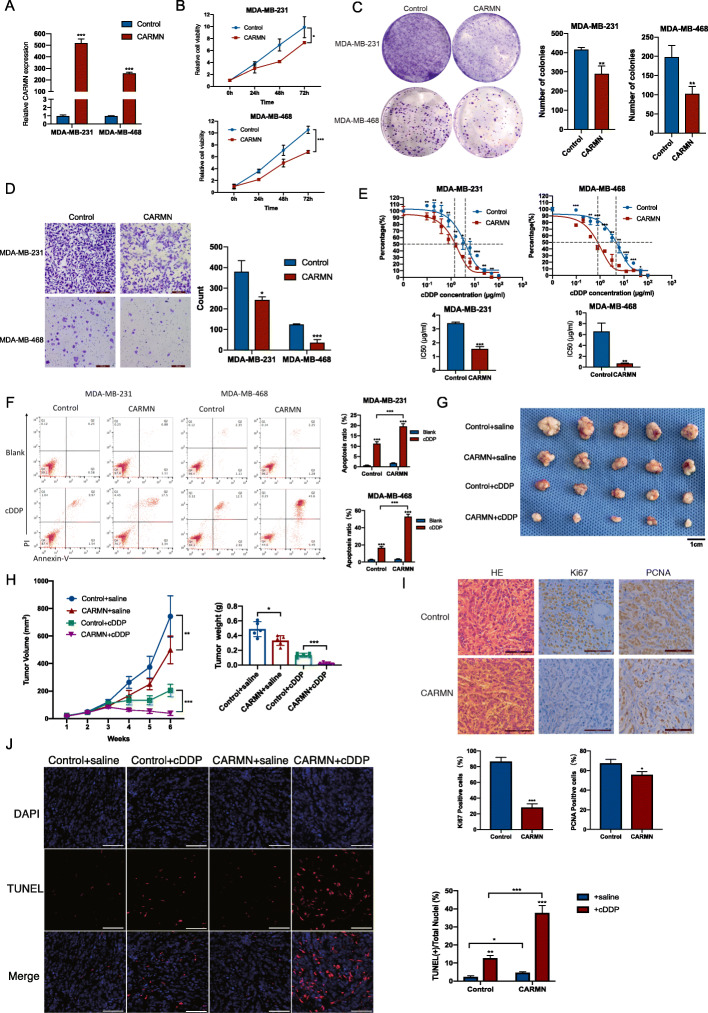
CARMN inhibits proliferation and sensitizes cisplatin in TNBC cells in vitro and in vivo. **A** Overexpression efficiency of CARMN confirmed by RT-qPCR. **B** Influence of CARMN overexpression on cell proliferation in TNBC cells detected by CCK8 assay. **C** Influence of CARMN overexpression on cell colony formation in TNBC cell. **D** Influence of CARMN overexpression on cell migration detected by transwell assays. **E** Effect of CARMN overexpression on sensitivity to cisplatin in TNBC cells reflected by relative activity of cells treated with different cisplatin concentration. **F** Cell apoptosis induced by cisplatin in TNBC cells overexpressing CARMN or control cells. **G**, **H** Xenograft tumors of 4 groups: CARMN overexpressed MDA-MB-468 cells + cisplatin; CARMN overexpressed MDA-MB-468 cells + saline; control MDA-MB-468 cells + cisplatin; control MDA-MB-468 + saline. **G** shows photos of xenograft tumors; **H** reflects volume and weight of xenograft tumors respectively. Scale bar = 1 cm. **I** HE staining and expression of Ki67 and PCNA in xenograft tumors with different expression of CARMN. Scale bar = 100 μm. **J** TUNEL assay of 4 groups of xenografts described above. Scale bar = 200 μm. IC50, 50% inhibition concentration; cDDP: cisplatin. Error bars represent means ± SD, *P < 0.05, **P < 0.01, ***P < 0.001

To elucidated CARMN’s role in triple negative breast cancer, CARMN expression and cisplatin sensitivity was detected in different breast cancer cell lines, including 4 TNBC cell lines (Figure S2A, S2B). In 4 TNBC cell lines, CARMN distributed mainly in nuclear, rather than in cytoplasm (Figure S2C). We overexpressed CARMN in TNBC cell lines MDA-MB-231 and MDA-MB-468 cells with relatively lower CARMN expression (Fig. 3A). CARMN overexpression significantly inhibited cell proliferation and colony formation of TNBC cells (Fig. 3B and C). Migration ability of cells was also obviously suppressed in CARMN overexpressed cells (Fig. 3D). Furthermore, CARMN overexpression caused more failure of cytokinesis, indicating more cancer cells failed in successfully separated into two cells [15] (Figure S2D). CARMN sensitized TNBC cells to cisplatin with lower IC50 (Fig. 3E). Apoptosis assay also demonstrated higher ratio of cell apoptosis after cisplatin treatment in CARMN upregulated cells (Fig. 3F).

We inhibited CARMN expression in TNBC cell lines Hs578T and BT20 with relatively higher CARMN level (Figure S2E). Inhibition of CARMN promoted TNBC proliferation (Figure S2F, S2G) and migration (Figure S2H). Cisplatin sensitivity also decreased following CARMN silence (Figure S2I) and lower rate of cell apoptosis was observed in CARMN inhibited cells (Figure S2J).

We injected MDA-MB-468 cells stably overexpressing CARMN or control cells into 5-week nude mouse flank and treated them with cisplatin or saline respectively. Firstly, we observed decrease in both tumor volume and tumor weight with CARMN overexpressed cells compared with control cells (Fig. 3G, H), with lower level of Ki67/PCNA positive rate (Fig. 3I) and higher level of TUNEL positive rate (Fig. 3J). Furthermore, cisplatin can reduce tumor growth more efficiently in CARMN overexpressed group than in control group (Fig. 3G, H). Comparison of xenografts treated with cisplatin showed significant elevation of TUNEL positive cells in CARMN overexpressed tumors (Fig. 3J).

### CARMN is correlated with various cisplatin resistance and cancer related pathways

**Fig. 4:**
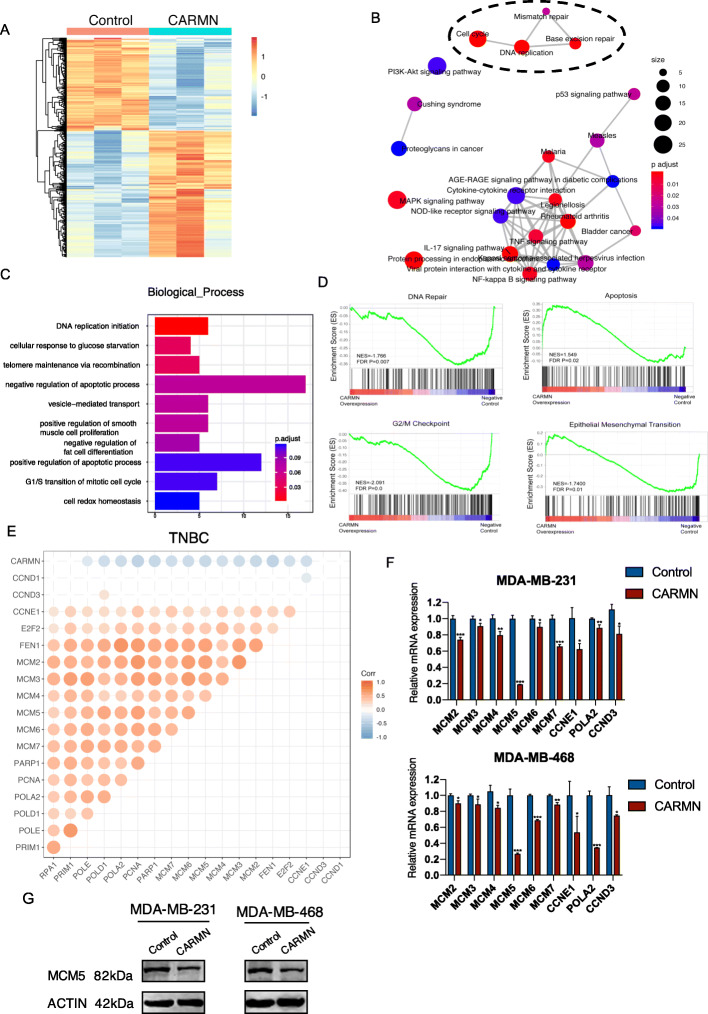
CARMN is related with cisplatin resistance and cancer related pathways in TNBC and dysregulates MCM5. **A** Heat map showing 638 differentially expressed genes (DEGs) according to RNA-seq of CARMN overexpressed MDA-MB-231 cells comparing with control cells. **B** Mapplot of KEGG pathway enrichment analysis of DEGs. Size of the dots represents number of DEGs enriched in each pathway and color of the dots represents *p* value of the pathways. **C** GO annotation in biological process enriched of DEGs. **D** GSEA enrichment analysis of CARMN regulated genes according to RNA-seq. **E** Correlation between CARMN and DNA replication and cell cycle related genes in TNBC of TCGA dataset. Positions lack of dots represent no statistically significant correlation between two genes. Color and size of dots represents correlation coefficient and larger dots represents higher correlation coefficient between two genes. **F** DNA replication related DEGs screened by RNA-seq were confirmed by RT-qPCR. **G** The effect of CARMN in MCM5 protein expression confirmed by Western Blot. EMT: epithelial-mesenchymal transition; cDDP: cisplatin. Error bars represent means ± SD, *P < 0.05, **P < 0.01, ***P < 0.001

RNA-seq of CARMN overexpressed and control MDA-MB-231 cells was applied to screen differentially expressed genes (DEGs) and potential target of CARMN. Under threshold of FDR < 0.05, fold change> 1.5, 638 DEGs were screened, including 262 downregulated genes and 376 upregulated genes (Fig. 4A, S3A, S3B). KEGG pathway enrichment of DEGs indicated CARMN participates in various pathways correlate with both cisplatin action and tumorigenesis including DNA replication, cell cycle, base excision repair, mismatch repair and microRNAs in cancer etc. (Fig. 4B, S3C, S3D). Statistical analysis showed DNA replication was the pathway with highest rich factor and showed globally downregulation in CARMN overexpressed cells. Besides KEGG, GO enrichment showed similar tendency, suggesting CARMN was related to pathways in cancer biological process in which DNA replication initiation was the most enriched pathway (Fig. 4C, S3F-S3H). GSEA enrichment analysis (Fig. 4D) demonstrated overexpression of CARMN caused downregulation of DNA repair, G2/M Checkpoint, Epithelial Mesenchymal Transition (EMT) and upregulation of apoptosis. Most DEGs in DNA replication, cell cycle and DNA repair pathways were downregulated when CARMN was overexpressed (Figure S4A).

DNA replication related DEGs were negatively correlated with CARMN expression in TCGA breast cancer cohort especially in TNBC cohort (Fig. 4E, Figure S4B). DNA replication DEGs were confirmed by RT-qPCR assay in CARMN overexpressed or control MDA-MB-231 and MDA-MB-468 cells and observed a general downregulation in these genes (Fig. 4F). Minichromosome maintenance complex component 5 (MCM5) was the most downregulated gene in both CARMN overexpressed MDA-MB-231 and MDA-MB-468 cells (Fig. 4F) which was also lower in protein level (Fig. 4G). After silencing CARMN expression, MCM5 significantly upregulated in Hs578T and BT20 cells in both mRNA (Figure S4C) and protein level (Figure S4D).

### CARMN inhibits DNA replication and regulates cell cycle in TNBC

**Fig. 5:**
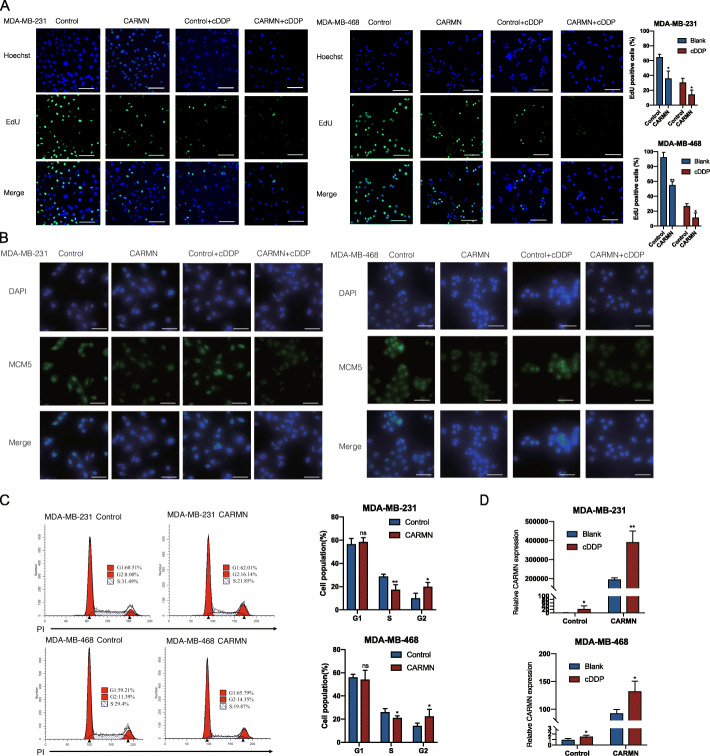
CARMN overexpression causes suppressed DNA replication and altered cell cycle distribution in TNBC. **A** EdU foci in CARMN overexpressed cells and control ones treat with cisplatin (5 μg/ml) or blank. Scale bar = 100 μm. **B** MCM5 immunofluorescent staining in TNBC cells overexpressing CARMN and control cells treated with or without cisplatin (5 μg/ml). Scale bar = 200 μm. **C** Effect of CARMN overexpression on cell cycle distribution of TNBC cells. **D** Effect of cisplatin (5 μg/ml) on CARMN expression in TNBC cells with or without CARMN overexpression. cDDP: cisplatin. Error bars represent means ± SD, *P < 0.05, **P < 0.01, ns: no significance

EdU foci was obviously decreased in CARMN overexpressed cells showing DNA replication activity is inhibited (Fig. 5A). In cells treated with cisplatin (5 μg/ml), EdU foci was also lower in CARMN overexpressing cells (Fig. 5A). MCM5 staining was also weaker in CARMN overexpressed cells than in control cells (Fig. 5B). In addition, CARMN overexpressing groups showed decreased percentage of S phase cells (Fig. 5C). Correspondingly, CARMN inhibited cells showed higher DNA replication activity (Figure S5A) and higher MCM5 fluorescence intensity (Figure S5B) in CARMN inhibited cells. CARMN silenced groups showed higher rate of S phase cells (Figure S5C).

After treated with cisplatin (5 μg/ml), CARMN is upregulated in all four TNBC cells (Fig. 5E, S5E). We conclude that CARMN can inhibit DNA replication activity and CARMN upregulation is feedback of cisplatin treatment.

### CARMN promotes cisplatin sensitivity through producing miR143-3p which targets MCM5

**Fig. 6:**
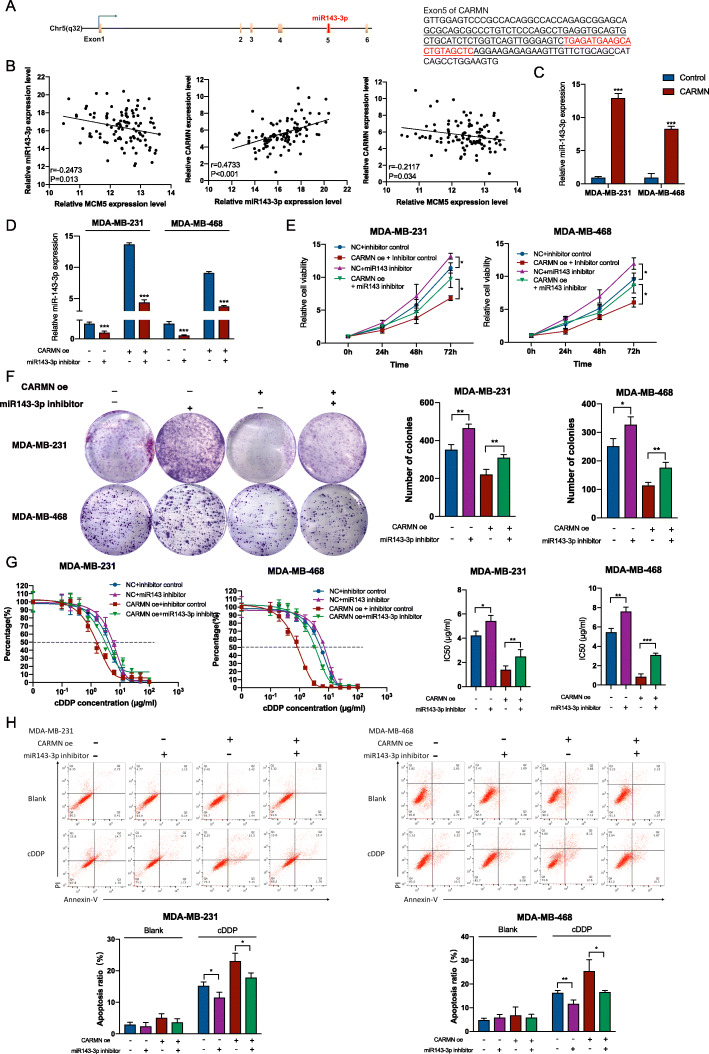
CARMN is host gene of miR143-3p which inhibits TNBC cell proliferation and promotes cisplatin sensitivity. **A** MiR143-3p is transcribed from exon5 of CARMN. Left panel shows the location of embedded miR143-3p in CARMN transcript unit. Right panel shows sequence of pre-miR143 (underline) and miR143-3p (red) embedded in sequence of CARMN exon5. **B** Correlation among miR143-3p, MCM5 and CARMN expression in TCGA TNBC samples. **C** Changes of miR143-3p expression in CARMN overexpression cells. **D** Efficiency of miR143-3p inhibitor in TNBC cells with or without CARMN overexpression. **D** Influence of CARMN exon5 overexpression on expression of miR143-3p confirmed with RT-qPCR. **E** Influence of miR143-3p inhibition on cell proliferation of CARMN overexpressed cells and control cells. **F** Influence of miR143-3p suppression on colony formation of CARMN overexpressed cells and control cells. **G** Effect of miR143-3p inhibition on sensitivity to cisplatin in CARMN overexpressed cells and control cells. **H** Effect of miR143-3p inhibition on cell apoptosis assays of CARMN overexpressed cells and control cells treated with cisplatin. CARMN oe: CARMN overexpression; cDDP: cisplatin; IC50, 50% inhibition concentration. Error bars represent means ± SD.*P < 0.05, **P < 0.01, ***P < 0.001

To further understand the potential effect of CARMN, we investigated the mechanism for dysregulation of MCM5 by CARMN. We predicted binding miRNAs and mRNAs of MCM5 and CARMN. In miRNAs binding with MCM5, we noticed miR143-3p which is embedded in CARMN (Supplementary Table 3), is predicted to combine 3’UTR of MCM5 according to both microT and miRanda [16–18]. Interestingly, in previous researches, CARMN was reported as the host gene of miR143 [6], suggesting that CARMN is the precursor of miR143 and could produce miR143. Through transcript sequence, oligonucleotide sequence of pre-miR143 and mature miR143-3p are confirmed to be embedded in exon5 of CARMN (Fig. 6A). There was a significant correlation among CARMN, miR143-3p and MCM5 expression in TCGA TNBC dataset (Fig. 6B). MiR143-3p was also remarkably upregulated in CARMN overexpressed cells (Fig. 6C). Given that published researches have already demonstrated miR143-3p can inhibit cell proliferation and sensitize cells to cisplatin [19, 20], CARMN possibly function partially by producing miR143-3p which can inhibit MCM5 by combining 3’UTR.

In TNBC cells with or without overexpressing CARMN, inhibition of miR143-3p (Fig. 6D) promoted cell proliferation and weakened suppression of cell proliferation by CARMN (Fig. 6E, F). In addition, inhibition of miR143-3p decreased cell sensitivity to cisplatin (Fig. 6G), with higher rate of cell apoptosis (Fig. 6H). Relationship between miR143-3p and CARMN was also demonstrated in CARMN silenced cells (Figure S6A). Overexpression miR143-3p (Figure S6B) in CARMN silenced or control cells led to inhibited proliferation ability (Figure S6C, S6D) and higher cisplatin sensitivity (Figure S6E, S6F).

**Fig. 7:**
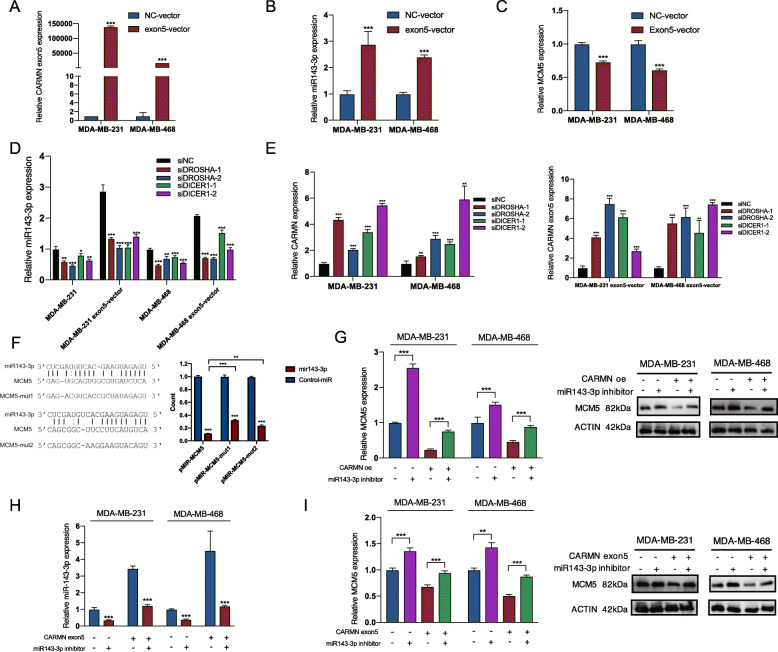
CARMN inhibits TNBC cell proliferation and promotes cisplatin sensitivity through producing miR143-3p which can combine and down regulate MCM5. **A** Efficiency of CARMN exon5 overexpression in TNBC cells. **B** Change of miR143-3p expression in CARMN exon5 overexpressed cells. **C** Change of MCM5 expression in CARMN exon5 overexpressed cells. **D** Influence of DROSHA and DICER1 suppression on miR143-3p level. **E** Effect of DROSHA and DICER1 suppression on CARMN expression (left) and CARMN exon5 (right). **F** Predicted binding site of miR143-3p and MCM5 (left). Luciferase reporter assay indicating binding ability of miR143-3p and 3’UTR of MCM5 in two predicted area (right). **G** Effect of miR143-3p inhibition on expression of MCM5 mRNA (left) and MCM5 protein (right) level in TNBC cells with or without CARMN overexpression. **H** Efficiency of miR143-3p inhibitor in TNBC cells with or without CARMN exon5 overexpression. **I** Effect of miR143-3p inhibition on expression of MCM5 mRNA (left) and MCM5 protein (right) level in TNBC cells with or without CARMN exon5 overexpression. cDDP: cisplatin. Error bars represent means ± SD, *P < 0.05, **P < 0.01, ***P < 0.001

Furthermore, to validate origin of miR143-3p, we overexpressed exon5 of CARMN (Fig. 7A) in which miR143-3p was embedded. MiR143-3p was obviously increased together with overexpression of CARMN exon5 (Fig. 7B), indicating that exon5 of CARMN was related with miR143-3p producing. MCM5 also showed lower expression in CARMN exon5 overexpressed cells (Fig. 7C).

Ribonuclease (RNase) III enzymes Drosha and Dicer are two important enzymes splicing precursor of miRNA and producing mature miRNA [21]. We silenced DROSHA and DICER1 (Dicer in *Homo sapiens*) in TNBC cell lines with or without CARMN exon5 overexpression (Figure S6G). Inhibited DROSHA and DICER1 decreased miR143-3p expression (Fig. 7D) and increased CARMN and CARMN exon5 expression (Fig. 7E), indicating miR143-3p production of CARMN and CARMN exon5 is Drosha and Dicer dependent.

To confirm combination of MCM5 with miR143-3p, we designed MCM5 mutated plasmid in 2 predicted combination area (Fig. 7F). Dual-luciferase assay confirmed combination of MCM5 and miR143-3p in both two predicted sites (Fig. 7F).

Combination between miR143-3p and MCM5 caused decreased MCM5 in both mRNA and protein level (Fig. 7G, S6H). MiR143-3p inhibition in CARMN exon5 overexpressed cells and control ones (Fig. 7H) also caused MCM5 upregulation (Fig. 7I).

To further confirm the production of miR143-3p of CARMN and CARMN exon5, we overexpressed CARMN in normal breast epithelium cell MCF10A (Figure S7A). We also discovered upregulated miR143-3p (Figure S7B) and inhibited MCM5 expression in CARMN overexpressed MCF10A (Figure S7C, S7D), which was also observed in CARMN exon5 transfected MCF10A cells (Figure S7E-S7H). Moreover, Exon5 of CARMN, in which miR143-3p was embedded, showed similar function of suppressing cell proliferation (Figure S8A, S8B) and sensitizing cisplatin as CARMN (Figure S8C, S8D).

Furthermore, we also suppressed MCM5 in MDA-MB-231 and MDA-MB-468 cells (Figure S9A), resulting inhibited cell proliferation (Figure S9B, S9C) and increased cisplatin sensitivity (Figure S9D).

As down-streams of CARMN, miR143-3p and MCM5 were also potential prediction factors for prognosis and cisplatin sensitivity in TNBC (Figure S10A-S10F).

## Discussion

With insufficient treatment targets, TNBC is more dependent on chemotherapy compared with the other subtypes of breast cancer, demanding precise biomarkers for suitable treatment strategy. Results in this research indicated CARMN is not only an efficient biomarker for identification of malignant transformation, but also a valuable predictive factor for prognosis and chemosensitivity for breast cancer. Our further exploration in molecular mechanism confirmed CARMN restoration as a potential target for anti-tumor and cisplatin resistance in TNBC.

Originally, CARMN exhibited essential role in modulating specification and differentiation of human cardiac precursor cell in published studies [6]. As a differentiation regulator, loss of CARMN can possibly lead to tumorigenesis and CARMN exhaustion is observed in many types of cancers. Although CARMN is a lncRNA significantly downregulated in TNBC, no research has mentioned CARMN’s role in TNBC. It is worth noting that we made the first demonstration that CARMN could suppress tumor malignant transformation, inhibit cancer cell proliferation and enhance cisplatin response synergistically in TNBC.

Our research firstly unmasked association between CARMN and cisplatin-based neoadjuvant chemotherapy response. Nearly half of the TNBC is able to obtain pathological complete response (pCR) after platinum-based neoadjuvant chemotherapy [22], which brings to significantly improved prognosis. Nevertheless, high rate of platinum resistance still limits clinical benefit from platinum-based neoadjuvant or adjuvant chemotherapy. Thus, identifying biomarkers for platinum sensitivity and target for elevating platinum sensitivity is now indispensable for TNBC treatment. Cisplatin causes DNA double strand breaks (DSB) which is mainly repaired through nucleotide excision repair (NER) during S phase [23]. RNA-Seq results showed CARMN was associated with cisplatin resistance and tumorigenesis pathways, which was in line with the results of in vivo and in vitro studies. Surprisingly, we discovered a global downregulation in MCM complex combined with MCM2–7, in which MCM5 was the lowest one. As evolutionary conserved proteins, MCM2–7 complex is recruited at origin recognition complex (ORC) region and act as a “license” of eukaryotes DNA replication, ensuring chromosome replication happen only one time in each cell cycle and undergo successful replication procedure [24]. As a member of MCM2–7 complex, persistent expression of MCM5 was reported to cause malign differentiation independently [25, 26]. Clinically, staining of MCM members was found to be obviously correlated with severity of dysplasia and was even reported to be better biomarker than widely used markers as PCNA and Ki67 [27, 28]. Consistently, Hang et al. also found high expression of all members of MCM complex in breast cancer was related with shorter survival time [29]. Despite initiating DNA replication, deficit of MCM complex, and also other DNA replication genes can cause replicative stress characterized with more DSBs and higher level of replicative fork instability. Elevated DSBs and replicative fork instability trigger elimination of cancer cells [30] and importantly enhance response of cytotoxic drugs interacting with DNA like platinum [31]. Thereby, we conclude exhaustion of DNA replication genes including MCM complex is one of the mechanisms explaining both enhanced cisplatin chemosensitivity and decreased proliferation ability in CARMN highly expressed cells. Despite MCM5 targeted by CARMN in current research, relationship between CARMN and the other members of MCM complex also need to be deeply explored in the future.

In our research is the first revealing regulation of MCM5 by CARMN was carried out by producing miR143-3p from exon5, which can target MCM5. According to previously studies about lncRNA and microRNA, lncRNAs are usually reported to act as competing endogenous RNAs (ceRNA) of miRNAs [32] while lncRNAs act as precursor or host gene of miRNA is relatively infrequent. Our results suggested CARMN acted as host gene of miR143-3p and functioned by upregulating derived miR143-3p. In genome location, majority of known mammalian miRNAs are within introns of protein-coding or non-coding transcription units (TU), and 10% are within exons of them, so called host gene [33]. Host genes undergo several procedures to successively produce embedded miRNA, including splicing of miRNA precursor or pri-miRNA by DROSHA in nuclear and splicing of pre-miRNA by Dicer in cytoplasm [34]. The relationship between function and regulation of miRNAs and their host genes is still ambiguous. There’s evidence that host genes may function partially by producing derived miRNAs. One reported example is miR675 which embedded in exon1 of its host gene lncRNA H19. H19 is found to be downregulated together with miR675 in prostate cancer and takes part in biological processes through miRNA’s combination with its downstream [35]. The same model can explain the role of CARMN in TNBC. We are the first to demonstrate miR143-3p is derived from exon5 of CARMN. Inhibition of miR143-3p can decrease the ability of CARMN in suppressing proliferation and chemotherapy sensitivity, by targeting an important DNA replication related gene MCM5. Thus, the relationship between host gene CARMN and miR143-3p mentioned in our research offers new evidence in understanding the mode of miRNAs and their host genes.

What further support our results is that the role of miR143 in suppressing different cancers including breast cancer has been reported previously through regulating various pathways [36–39]. In terms of drug sensitivity, miR143-3p was also reported to sensitize cisplatin and inhibit tumor progression in gastric cancer [19]. Our results verify chemotherapy sensitizing and anti-tumor function of miR143-3p in TNBC and we also identified MCM5 as a new target of miR143-3p.

There still exists several limitations in our research. First, our RNA-seq showed CARMN may potentially regulated many cancer-related genes and pathways in which we only explored the most enriched pathway DNA replication and DNA replication related gene MCM5. Other possible down stream of CARMN will be further studied in the future. Secondly, our patients administrated cisplatin-paclitaxel regimens, while in experiment in vitro and in vivo we only verified the association between cisplatin and CARMN. Thus, the role of CARMN on paclitaxel or cisplatin plus paclitaxel should be explored in the future.

## Conclusions

**Fig. 8:**
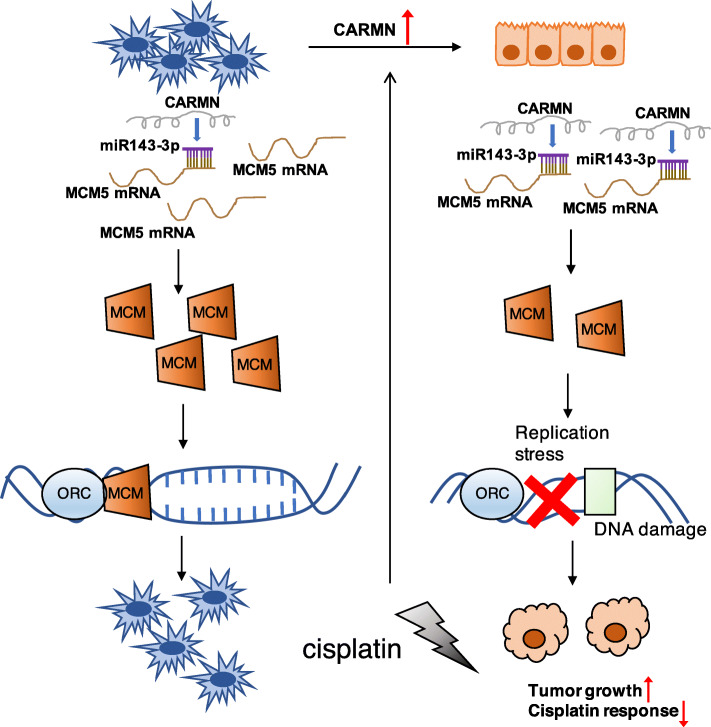
Schema of CARMN’s role in triple negative breast cancer: Triple negative breast cancer cells have low level of CARMN. CARMN can inhibit breast cancer and sensitize chemotherapy by regulating many essential pathways and processes. CARMN functions partially by producing embedded miR143-3p which binds with MCM5. MCM5 acts as member of DNA replication initiation factor MCM complex, which combines with ORC and licenses DNA replication. Suppressing of MCM5 by CARMN inhibits normal DNA replication, leading to inhibited tumor growth and higher cisplatin sensitivity. Cisplatin can upregulate CARMN to cause cell apoptosis as a feedback. cDDP: cisplatin; ORC: origin recognition complex

In conclusion, we initially elucidated lncRNA CARMN could generate better response to cisplatin-contained neoadjuvant chemotherapy in TNBC. According to experiments in vivo and in vitro, CARMN enhances cisplatin sensitivity and inhibits cell proliferation in TNBC. CARMN participates in cisplatin resistance and tumorigenesis related pathways, especially DNA replication. It is further confirmed that high CARMN expression suppresses DNA replication activity. CARMN is also increased as feedback of cisplatin treatment. CARMN sensitizes cisplatin and represses development of TNBC through increasing miR143-3p as its host gene, and miR143-3p targets at a crucial DNA replication licenser MCM5, which may cause abnormal DNA replication (Fig. 8). Our results provide restoration of CARMN as potential therapeutic target and prognostic predicting marker for TNBC.

## Supplementary Information

**Additional file 1: Supplementary Figure 1.** CARMN is a valuable biomarker in distinguishing tumor from normal tissue. **Supplementary Figure 2.** CARMN regulates cell proliferation and cisplatin sensitivity of TNBC. **Supplementary Figure 3.** RNA-seq indicates CARMN participating in essential cancer related pathways. **Supplementary Figure 4.** CARMN participates in DNA replication and downregulates DNA replication related genes. **Supplementary Figure 5.** CARMN suppression causes suppressed DNA replication and altered cell cycle distribution in TNBC. **Supplementary Figure 6.** MiR143-3p inhibits proliferation and cisplatin sensitivity in TNBC. **Supplementary Figure 7.** Confirmation of CARMN/miR143-3p/MCM5 axis in normal breast epithelium cell line MCF10A. **Supplementary Figure 8.** CARMN exon5 inhibits TNBC cell proliferation and promotes cisplatin sensitivity. **Supplementary Figure 9.** MCM5 promotes TNBC cell proliferation and decreases cisplatin sensitivity. **Supplementary Figure 10.** MiR143-3p and MCM5 are both prognostic factors of breast cancer and TNBC.

**Additional file 2: Supplementary Table 1.** Primers of qRT-PCR. **Supplementary Table 2.** Sequence of siRNAs, miR143-3p inhibitor and miR-143-3p mimics. **Supplementary Table 3.** miRNAs predicted to bind with MCM5.

**Additional file 3.**

## Data Availability

TCGA dataset used in the current study can be accessed from: https://portal.gdc.cancer.gov. GEO datasets used in the current study can be accessed from: https://www.ncbi.nlm.nih.gov. RNA-seq data are not publicly available yet, but can be available from the corresponding author, Dr. Jinsong Lu (email address: lujjss@163.com).

## Abbreviations

TNBC: Triple negative breast cancer
lncRNAs: Long noncoding RNAs
CARMN: Cardiac Mesoderm Enhancer-associated Noncoding RNA
TCGA: The Cancer Genome Atlas
cDDP: Cisplatin
MCM: Minichromosome Maintenance Proteins
OR: Odds ratio
95% CI: 95% confidence interval
pCR: Pathological complete response
ROC: Receiver operating characteristic
AUC: Area under curve
