# Supplementary material for: LncRNA CARMN overexpression promotes prognosis and chemosensitivity of triple negative breast cancer via acting as miR143-3p host gene and inhibiting DNA replication
Source: J Exp Clin Cancer Res. 2021 Jun 23;40:205. doi: 10.1186/s13046-021-02015-4 (PMC8220716; doi:10.1186/s13046-021-02015-4)
Supplement: Supplementary file 1 — Additional file 1: Supplementary Figure 1. CARMN is a valuable biomarker in distinguishing tumor from normal tissue. Supplementary Figure 2. CARMN regulates cell proliferation and cisplatin sensitivity of TNBC. Supplementary Figure 3. RNA-seq indicates CARMN participating in essential cancer related pathways. Supplementary Figure 4. CARMN participates in DNA replication and downregulates DNA replication related genes. Supplementary Figure 5. CARMN suppression causes suppressed DNA replication and altered cell cycle distribution in TNBC. Supplementary Figure 6. MiR143-3p inhibits proliferation and cisplatin sensitivity in TNBC. Supplementary Figure 7. Confirmation of CARMN/miR143-3p/MCM5 axis in normal breast epithelium cell line MCF10A. Supplementary Figure 8. CARMN exon5 inhibits TNBC cell proliferation and promotes cisplatin sensitivity. Supplementary Figure 9. MCM5 promotes TNBC cell proliferation and decreases cisplatin sensitivity. Supplementary Figure 10. MiR143-3p and MCM5 are both prognostic factors of breast cancer and TNBC. [file 13046_2021_2015_MOESM1_ESM.docx]

**
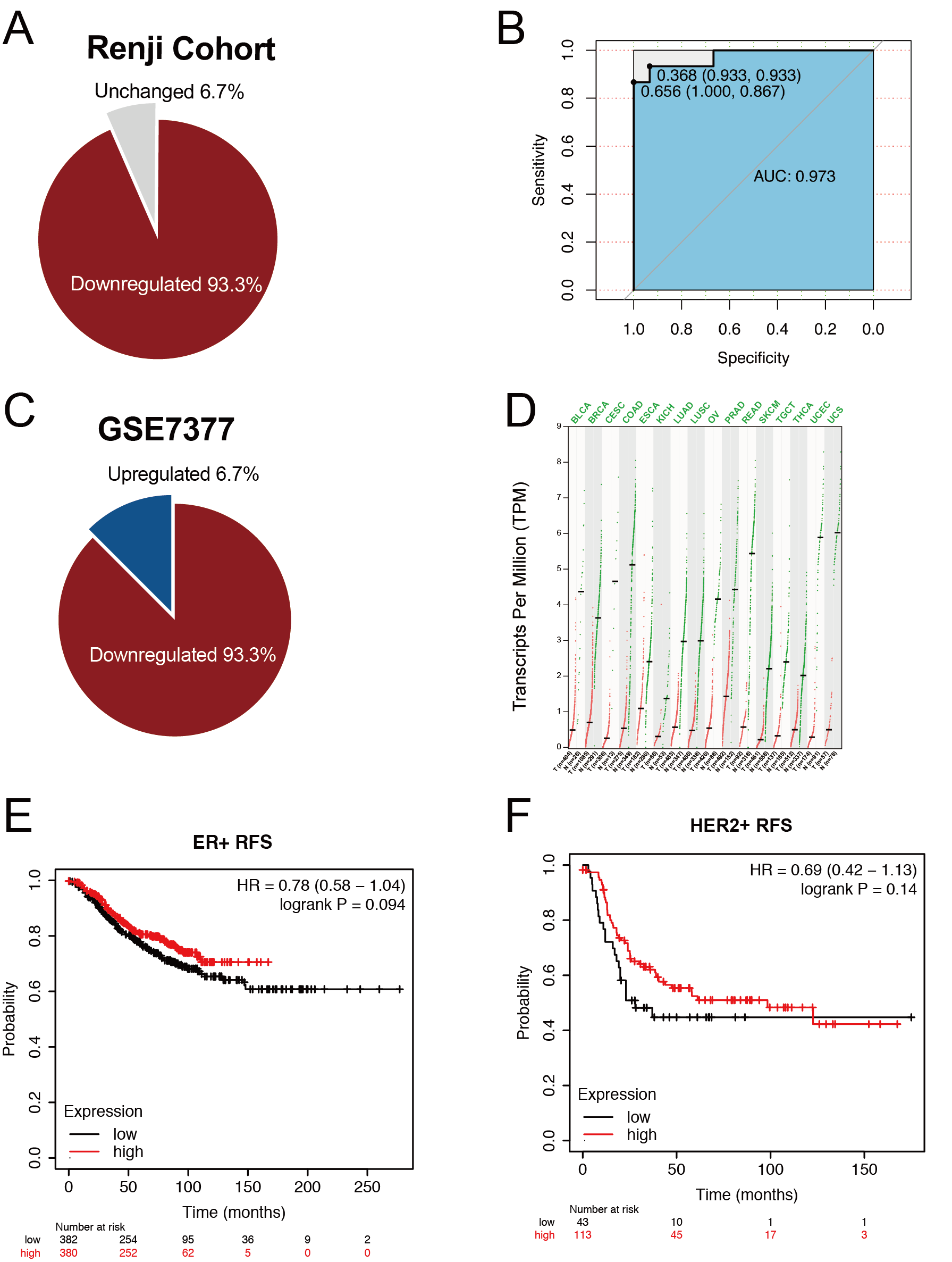
**

**Supplementary Figure 1: CARMN is a valuable biomarker in distinguishing tumor from normal tissue.**

**(A)** Distribution of changing tendency of CARMN in breast cancer tissue comparing with normal tissue. **(B)** ROC curve of CARMN acting as a predictor in distinguishing breast cancer from normal ones in Renji cohort. AUC is used for evaluating performance of CARMN expression. **(C)** Distribution of changing tendency of CARMN in hyperplastic enlarged lobular units (HELU) comparing with paired terminal duct lobular units (TDLU). **(D)** Besides breast cancer, CARMN is deregulated in many types of malignant tumor according to TCGA database. T: Tumor, N: Normal. Abbreviations of malignant tumors in green indicates statistical significance. **(E), (F)** RFS of patients with different expression of CARMN in ER+ or HER2+ breast cancer according to Kaplan-Meier Plotter. NAC: Neoadjuvant Chemotherapy; ROC: Receiver Operating Characteristic; AUC: Area under curve.


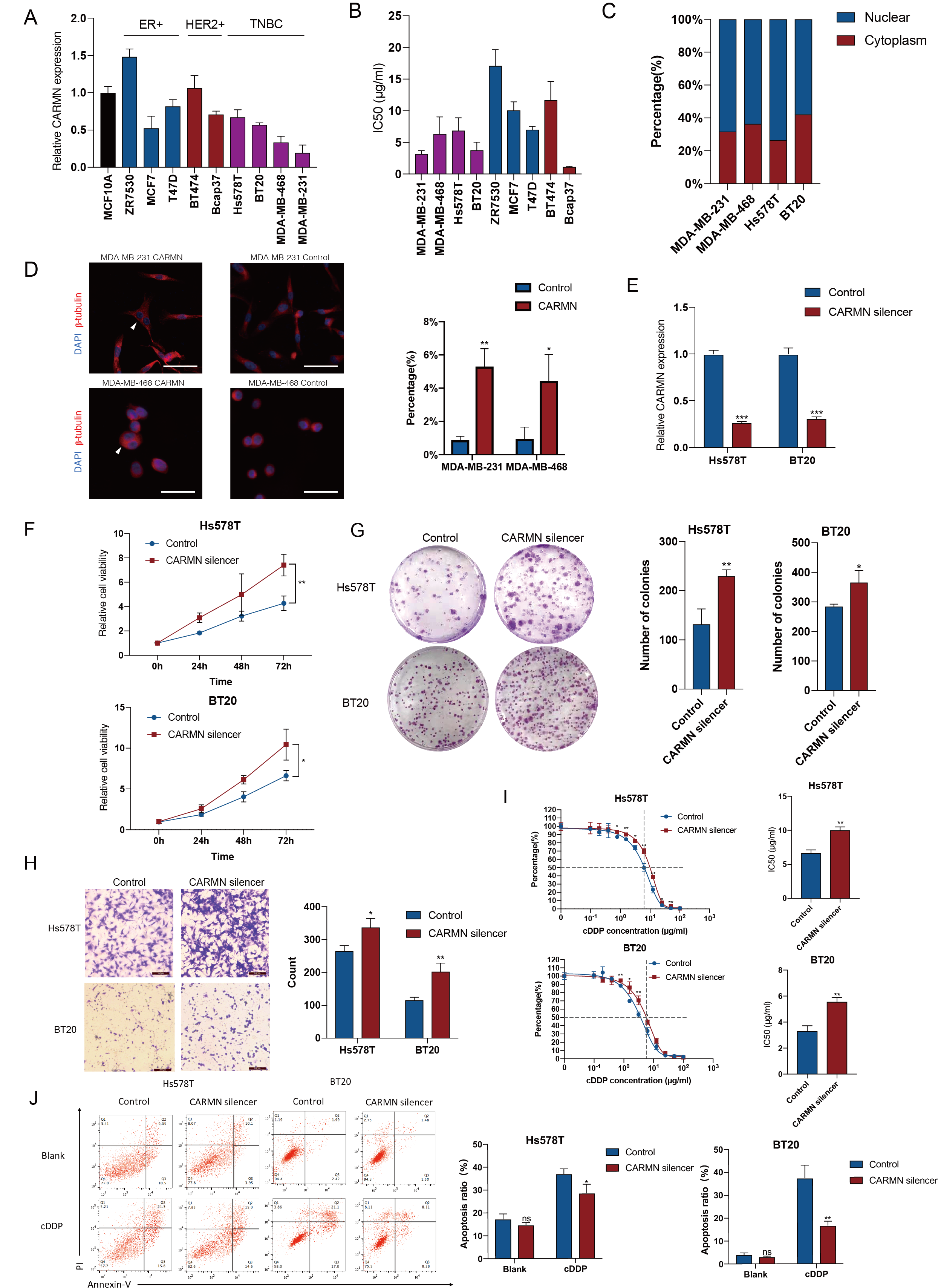


**Supplementary Figure 2: CARMN regulates cell proliferation and cisplatin sensitivity of TNBC.**

**(A)** CARMN expression in different breast cancer cell lines and in normal breast epithelium cell MCF10A. **(B)** IC50 of cisplatin in different breast cancer cell lines. **(C)** CARMN sublocalization in TNBC cell lines. **(D)** Effect of CARMN overexpression on cytokinesis detected by immunofluorescence of DAPI and β-tubulin. Immunofluorescence images in left panel show typical binucleation (white arrow) which represents unstably replicated cells. right panel shows statistical analysis of binucleation ratio in different cells. Scale bar = 50μm. **(E)** Efficiency of CARMN inhibition confirmed by RT-qPCR. **(F)** Influence of CARMN inhibition on cell proliferation in TNBC cells detected by CCK8 assay. **(G)** Influence of CARMN inhibition on cell colony formation in TNBC cell. **(H)** Influence of CARMN inhibition on cell migration detected by transwell assays. **(I)** Effect of CARMN inhibition on sensitivity to cisplatin in TNBC cells. **(J)** Cell apoptosis induced by cisplatin in TNBC cells with inhibited CARMN or control cells. IC50, 50% inhibition concentration. Error bars represent means ± SD, *P<0.05, **P<0.01, ***P<0.001.


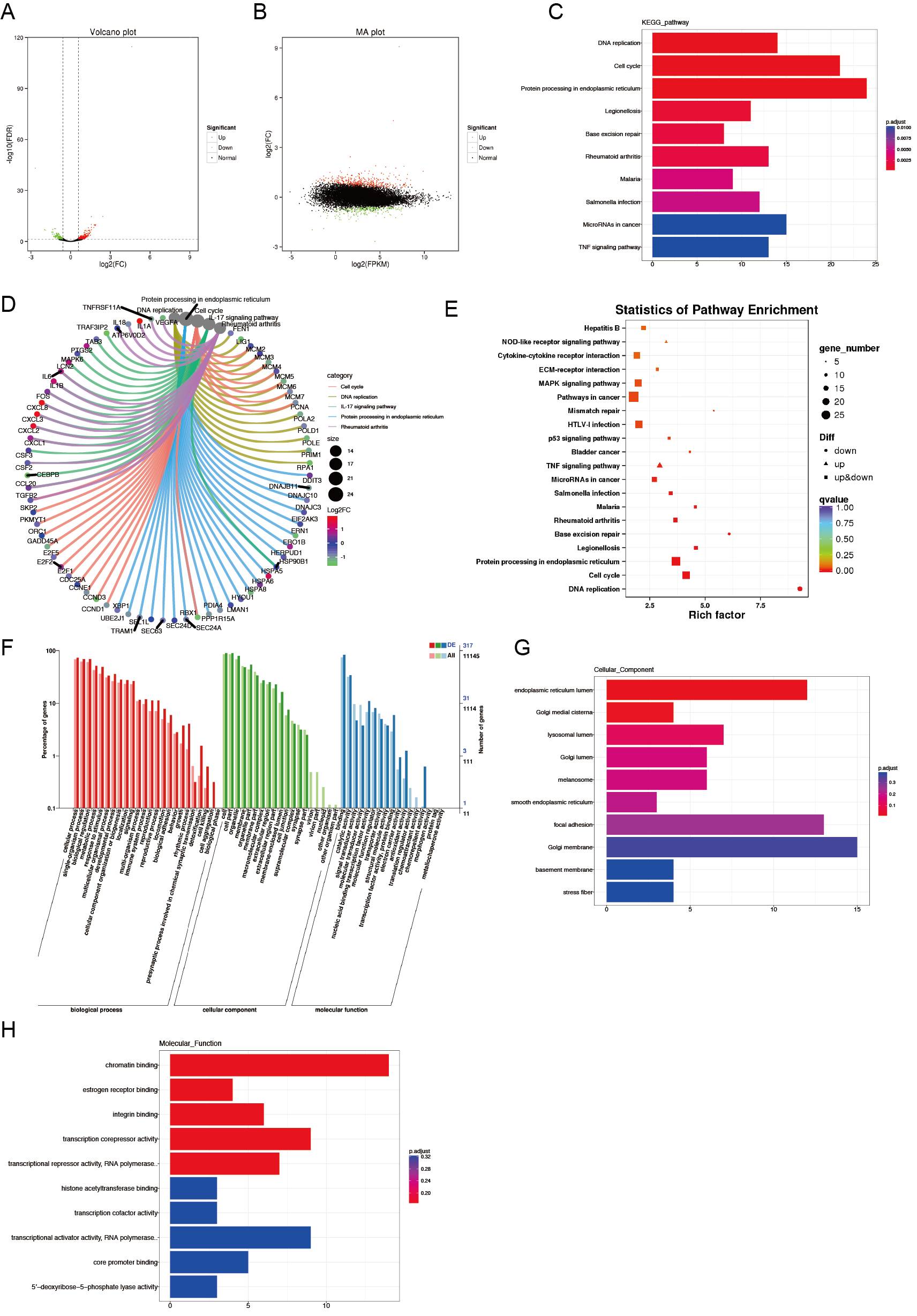


**Supplementary Figure 3: RNA-seq indicates CARMN participating in essential cancer related pathways.**

**(A), (B)** Volcano plot **(A)** and M-versus-A (MA) plot **(B)** of differentially expressed genes (DEGs) in CARMN overexpressed MDA-MB-231 cells, with threshold of |Fold change|>1.5, FDR<0.05. **(C), (D)** KEGG pathway enrichment results of DEGs in CARMN overexpressed cells. **(E)** Statistics of KEGG pathway enrichment showing detail regulatory condition of enriched pathways of DEGs. **(F)-(H)** GO annotation analysis of DEGs. **(F)** shows GO annotation distribution of all genes and DEGs, in which annotations with significant difference between all genes and DEGs are potentially regulated by CARMN. **(G)** and **(H)** are GO annotation analysis in different classifications including cellular component and molecular function in detail.


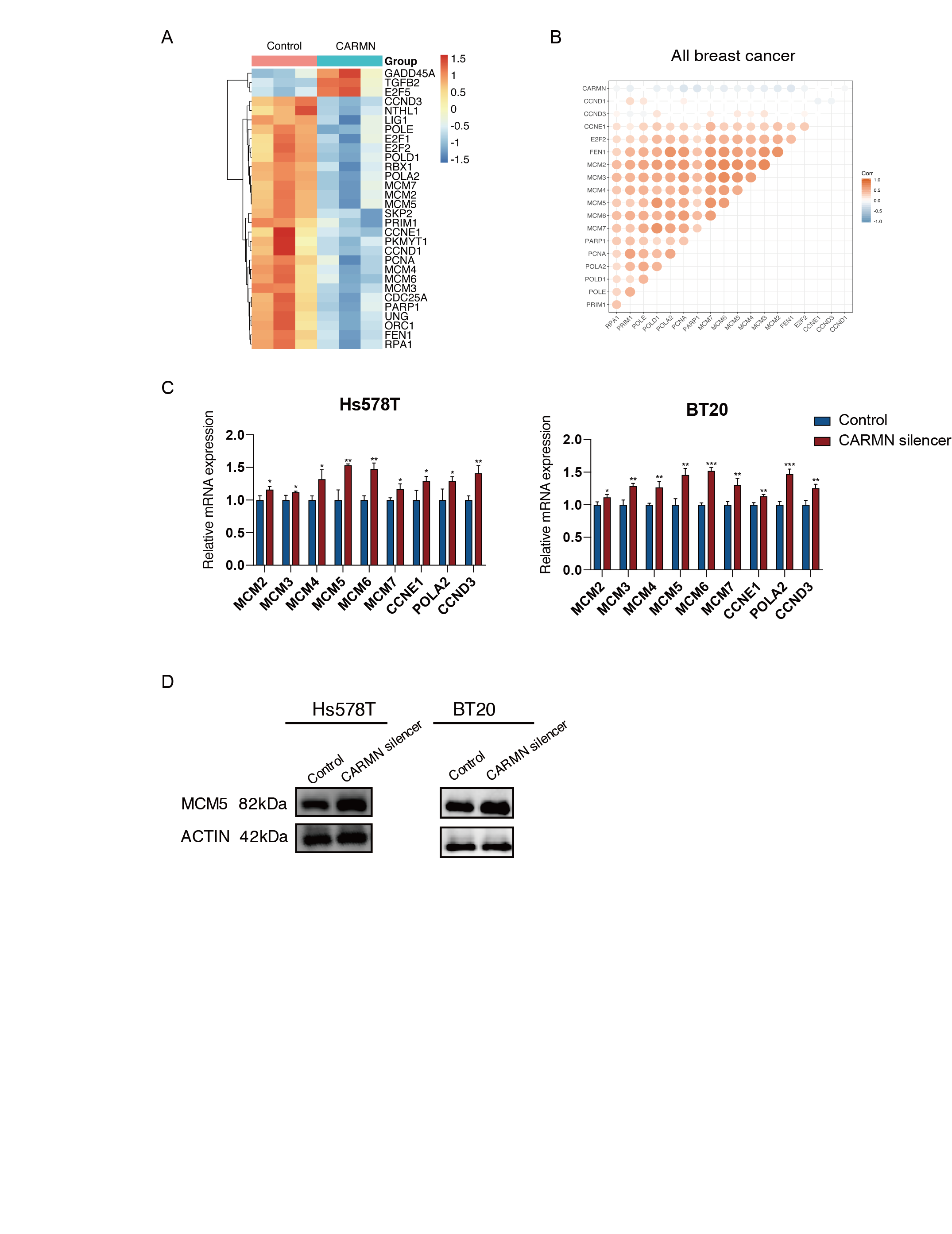


**Supplementary Figure 4: CARMN participates in DNA replication and downregulates DNA replication related genes.**

**(A)** Heat map showing DEGs participating in DNA replication, cell cycle and DNA repair pathways. **(B)** Correlation heat map of CARMN and DNA replication and cell cycle related genes in all breast cancer in TCGA dataset. Exhibited dots are data with statistical significance. **(C)** DNA replication related DEGs screened by RNA-seq were confirmed by RT-qPCR in CARMN silenced cells. **(D)** The effect of CARMN inhibition in MCM5 protein expression confirmed by Western Blot. Error bars represent means ± SD, *P<0.05, **P<0.01, ***P<0.001.


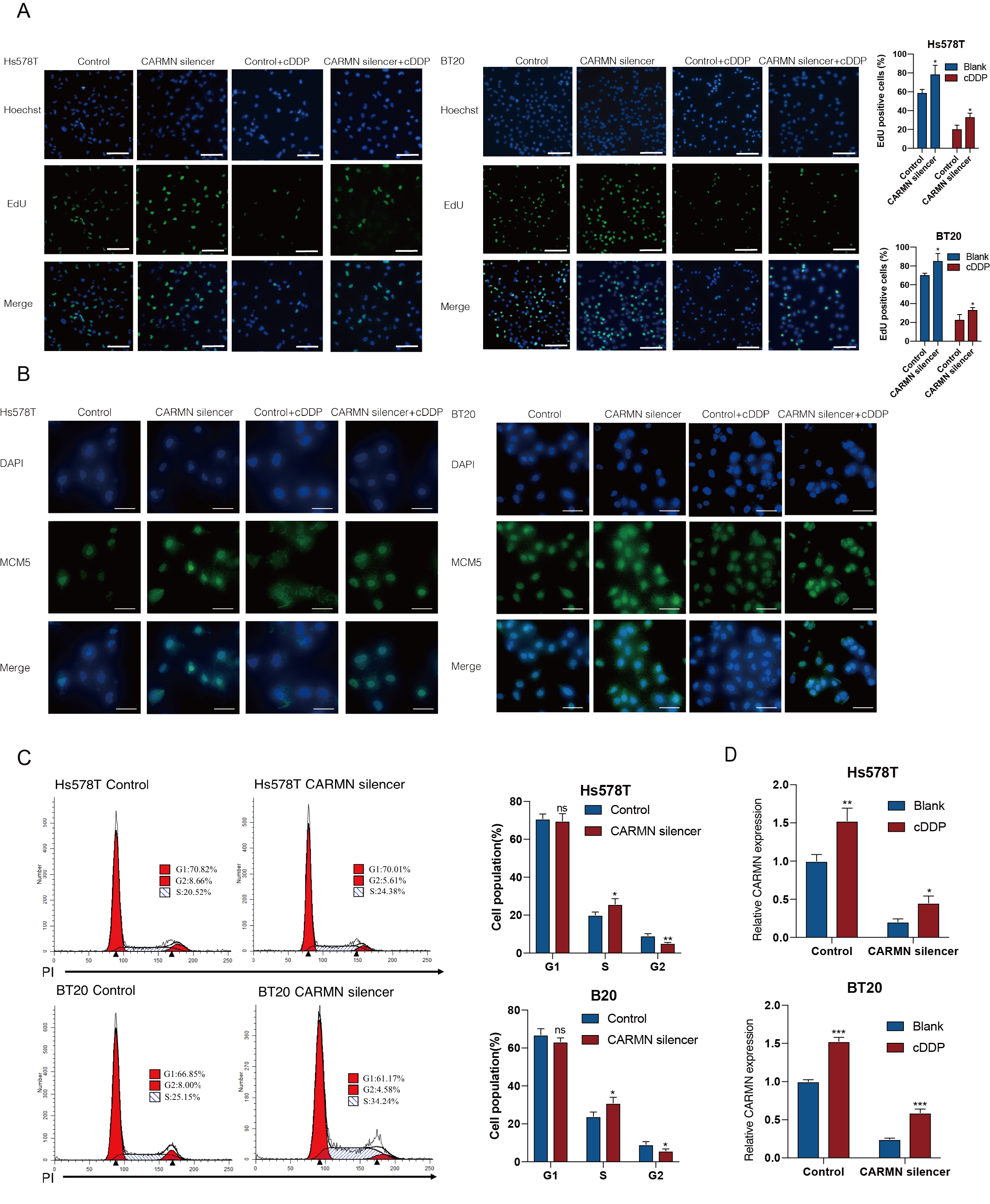


**Supplementary Figure 5: CARMN suppression causes suppressed DNA replication and altered cell cycle distribution in TNBC.**

**(A)** EdU foci in CARMN silenced cells and control ones treat with cisplatin (5μg/ml) or blank. Scale bar = 100μm. **(B)** MCM5 staining in TNBC cells with inhibited CARMN and control cells treat with cisplatin (5μg/ml) or blank. Scale bar = 200μm. **(C)** Effect of CARMN suppression on cell cycle distribution of TNBC cells. **(D)** Effect of cisplatin (5μg/ml) on CARMN expression in TNBC cells with or without CARMN suppression. cDDP: cisplatin. Error bars represent means ± SD, *P<0.05, **P<0.01, ns: no significance.


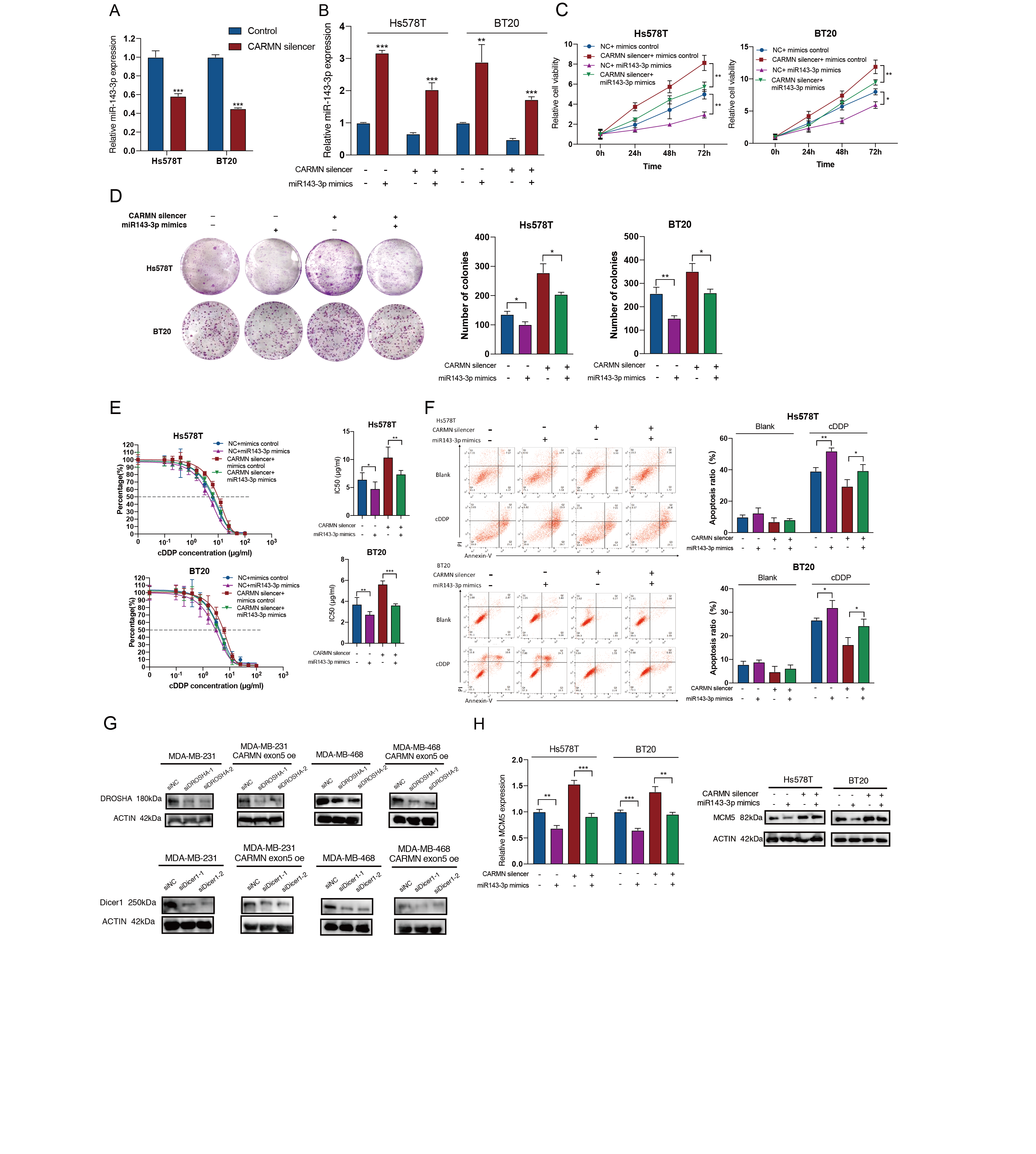


**Supplementary Figure 6: MiR143-3p inhibits proliferation and cisplatin sensitivity in TNBC.**

**(A)** MiR143-3p expression in CARMN silenced TNBC cells or control cells. **(B)** Efficiency of miR143-3p overexpression in TNBC cells with or without CARMN inhibition. **(C)** Influence of miR143-3p mimics on cell proliferation of CARMN suppressed cells and control cells. **(D)** Influence of miR143-3p mimics on colony formation of CARMN overexpressed cells and control cells. **(E)** Effect of miR143-3p mimics on sensitivity to cisplatin in CARMN silenced cells. **(F)** Effect of miR143-3p inhibition on cell apoptosis assays of CARMN overexpressed cells and control cells treated with cisplatin. **(G)** Efficiency of DROSHA (left) and DICER1 (right) inhibition in TNBC cells with or without CARMN exon5 overexpression. **(H)** Influence of miR143-3p overexpression on MCM5 mRNA level (left) and protein level (right) in CARMN silenced cells or control ones. cDDP: cisplatin; IC50, 50% inhibition concentration. Error bars represent means ± SD.*P<0.05, **P<0.01, ***P<0.001.


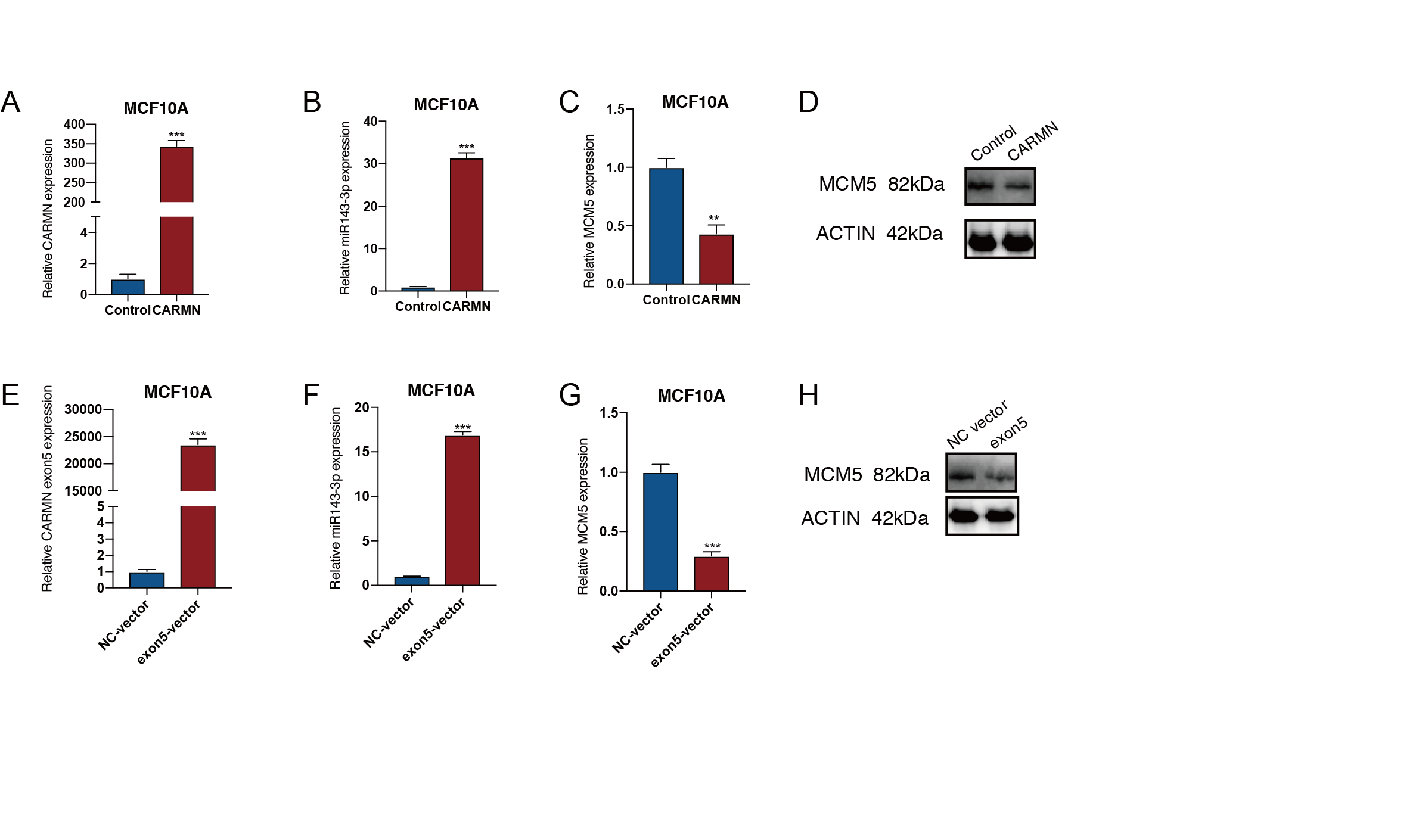


**Supplementary Figure 7: Confirmation of CARMN/miR143-3p/MCM5 axis in normal breast epithelium cell line MCF10A.**

**(A)** Efficiency of CARMN overexpression in MCF10A. **(B)** Influence of CARMN overexpression in miR143-3p level in MCF10A. **(C)** Influence of CARMN overexpression in MCM5 mRNA level in MCF10A. **(D)** Influence of CARMN overexpression in MCM5 protein level in MCF10A. **(E)** Efficiency of CARMN exon5 overexpression in MCF10A. **(F)** Influence of CARMN exon5 overexpression in miR143-3p level in MCF10A. **(G)** Influence of CARMN exon5 overexpression in MCM5 mRNA level in MCF10A. **(H)** Influence of CARMN exon5 overexpression in MCM5 protein level in MCF10A. Error bars represent means ± SD.*P<0.05, **P<0.01, ***P<0.001.


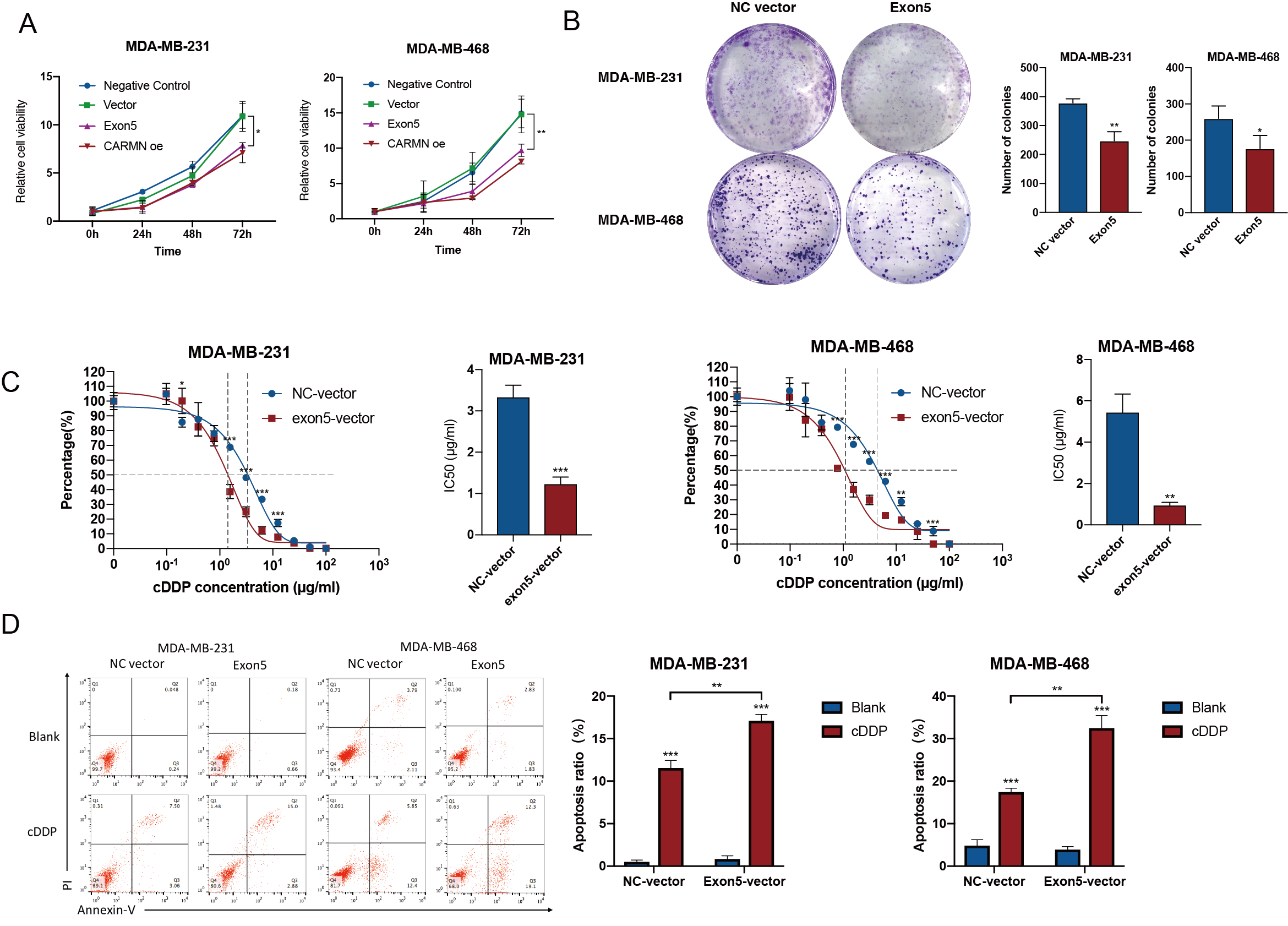


**Supplementary Figure 8: CARMN exon5 inhibits TNBC cell proliferation and promotes cisplatin sensitivity.**

**(A), (B)** Cell proliferation **(A)** detected by CCK8 and colony formation assay **(B)** of TNBC cells in 4 groups: control, control vector, CARMN exon5 vector and CARMN overexpression. **(C)** Effect of CARMN exon5 overexpression on sensitivity to cisplatin in TNBC cells. **(D)** Effect of CARMN exon5 overexpression on cell apoptosis assays of TNBC cells treated with cisplatin. CARMN oe: CARMN overexpression; cDDP: cisplatin; IC50, 50% inhibition concentration. Error bars represent means ± SD.*P<0.05, **P<0.01, ***P<0.001


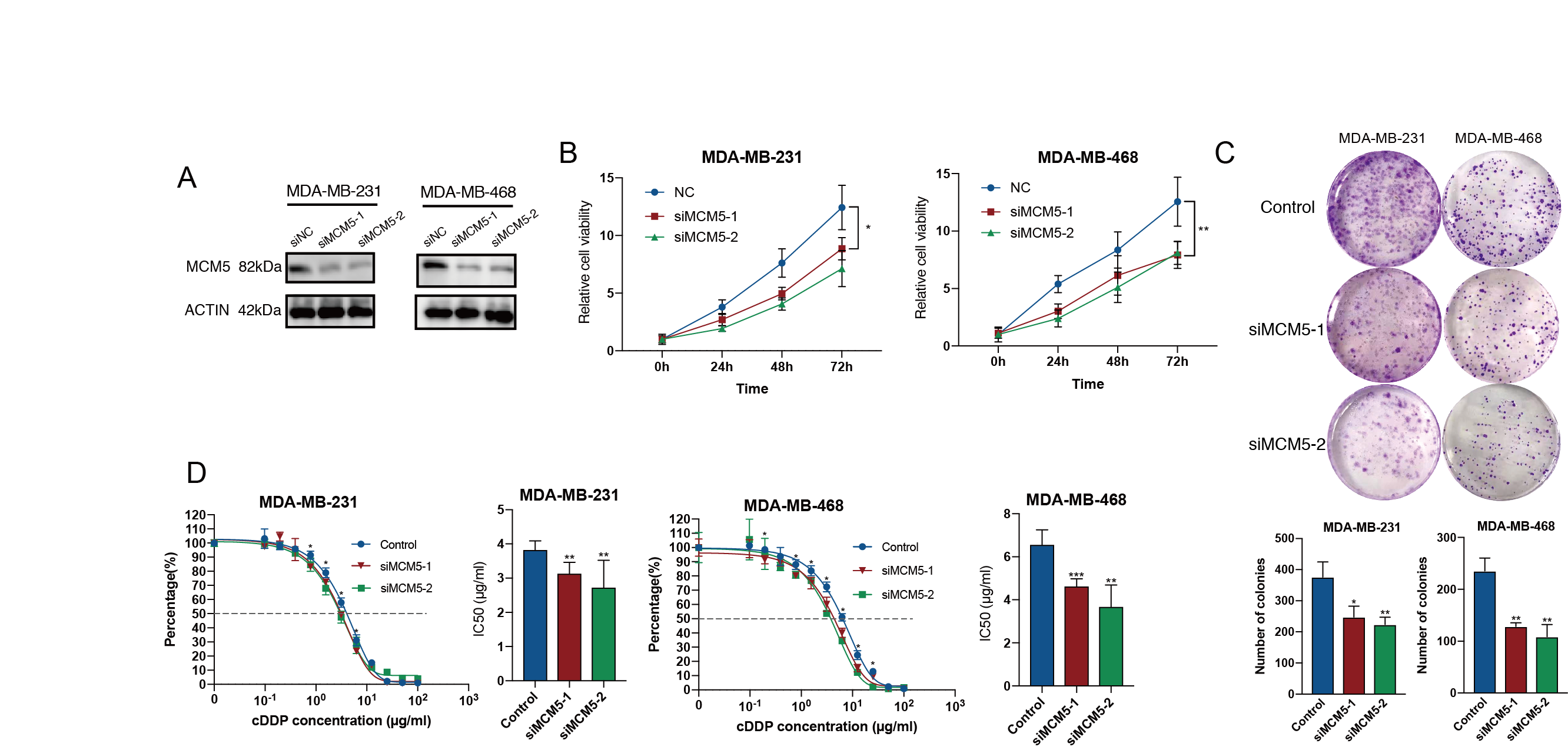


**Supplementary Figure 9: MCM5 promotes TNBC cell proliferation and decreases cisplatin sensitivity.**

**(A)** Efficiency of MCM5 suppression in TNBC cell lines. **(B)** Effect of MCM5 suppression on cell proliferation. **(C)** Effect of MCM5 suppression on colony formation of TNBC cells. **(D)** Influence of MCM5 suppression on cisplatin sensitivity in TNBC cells. cDDP: cisplatin; IC50, 50% inhibition concentration. Error bars represent means ± SD.*P<0.05, **P<0.01, ***P<0.001


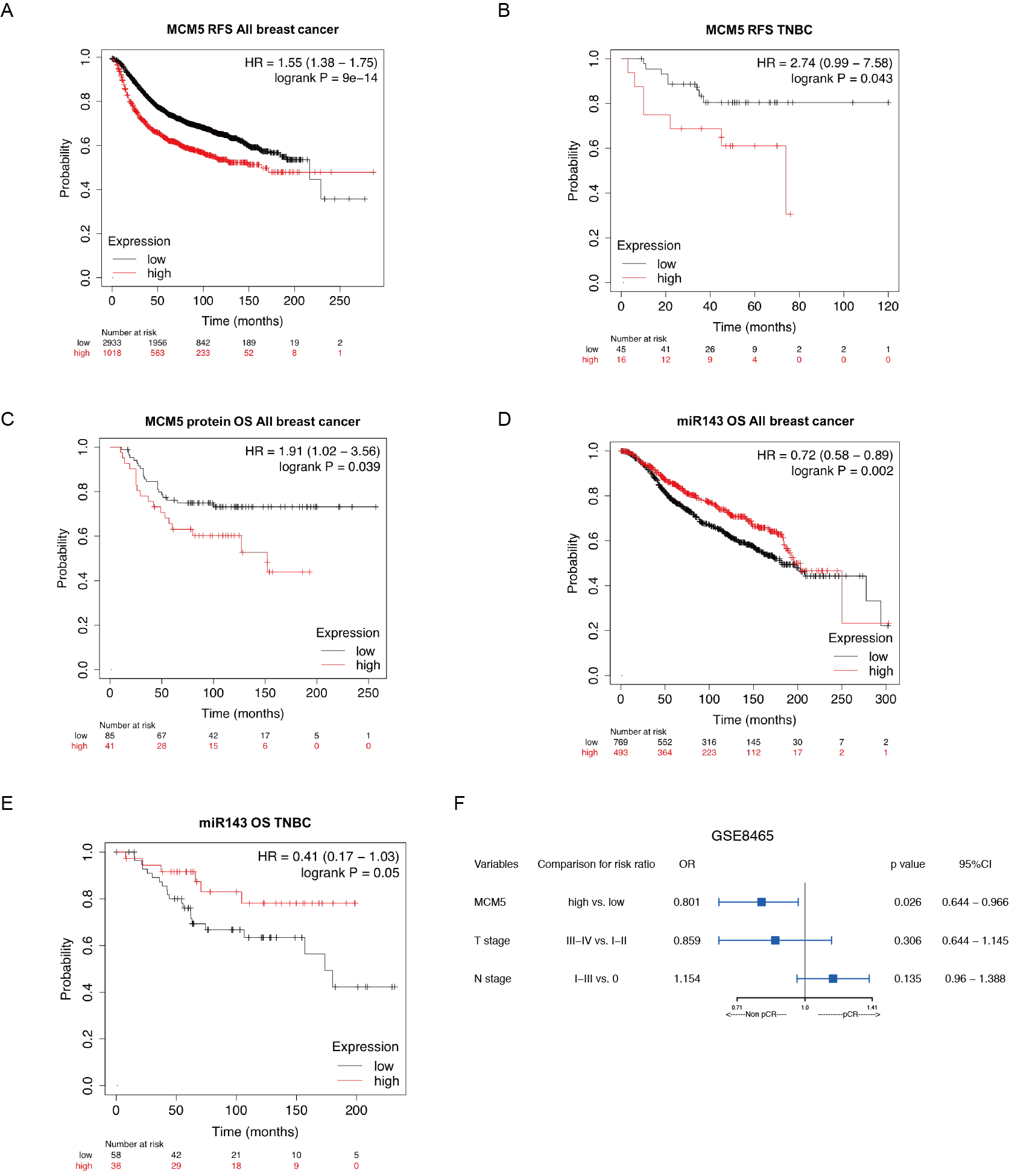


**Supplementary Figure 10: MiR143-3p and MCM5 are both prognostic factors of breast cancer and TNBC.**

**(A), (B)** RFS of all breast cancer **(A)** patients or TNBC **(B)** with different expression of MCM5 according to Kaplan-Meier Plotter. **(C)** RFS of all breast cancer patients with different expression of MCM5 protein according to Kaplan-Meier Plotter. **(D), (E)** OS of all breast cancer **(D)** patients or TNBC **(E)** with different expression of miR143-3p according to Kaplan-Meier Plotter. **(F)** MCM5 is negatively related with response of neoadjuvant chemotherapy containing cisplatin according to multivariate logistics regression analysis of GSE8465. RFS: Relapse-free survival; OS: Overall survival; pCR, pathological complete response; OR, odd ratio.
