# Supplementary material for: LncRNA CARMN overexpression promotes prognosis and chemosensitivity of triple negative breast cancer via acting as miR143-3p host gene and inhibiting DNA replication
Source: J Exp Clin Cancer Res. 2021 Jun 23;40:205. doi: 10.1186/s13046-021-02015-4 (PMC8220716; doi:10.1186/s13046-021-02015-4)
Supplement: Supplementary file 2 — Additional file 2: Supplementary Table 1. Primers of qRT-PCR. Supplementary Table 2. Sequence of siRNAs, miR143-3p inhibitor and miR-143-3p mimics. Supplementary Table 3. miRNAs predicted to bind with MCM5. [file 13046_2021_2015_MOESM2_ESM.docx]

**Supplementary Tables**

**Supplementary Table 1: Primers of qRT-PCR**

| CARMN | Forward | 5'-AGGGCCAGCAGCAGGC-3' |
| --- | --- | --- |
|  | Reverse | 5'-TCAGGAAATGTCTCTGGCTGTG-3' |
| β-actin | Forward | 5'-CATGTACGTTGCTATCCAGGC-3' |
|  | Reverse | 5'-CTCCTTAATGTCACGCACGAT-3' |
| RPA1 | Forward | 5'-GCTCATGAGTGATGGATTGAAC-3' |
|  | Reverse | 5'-AGTTGCTGGACAATTGTTCTTC-3' |
| PARP1 | Forward | 5'-AAGCTTGAAAAAGCCCTAAAGG-3' |
|  | Reverse | 5'-CTGCTTGTTGAAGATGAGTAGC-3' |
| CCND3 | Forward | 5'-CTTACTGGATGCTGGAGGTATG-3' |
|  | Reverse | 5'-GTAGCGATCCAGGTAGTTCATG-3' |
| CCNE1 | Forward | 5'-TTGTGTCCTGGCTGAATGTATA-3' |
|  | Reverse | 5'-AAGGAAATTCAAGGCAGTCAAC-3' |
| MCM7 | Forward | 5'-TGCAAGAACATAGTGATCAGGT-3' |
|  | Reverse | 5'-GTTCATCTTCACAATCCGATGG-3' |
| PCNA | Forward | 5'-TAATTTCCTGTGCAAAAGACGG-3' |
|  | Reverse | 5'-AAGAAGTTCAGGTACCTCAGTG-3' |
| MCM3 | Forward | 5'-CTTCTAATAGGAGACCCATCCG-3' |
|  | Reverse | 5'-AATTCATCAATGCAAACCACGC-3' |
| MCM4 | Forward | 5'-ATCTCCCTCTCAGAGACGTAG-3' |
|  | Reverse | 5'-TGTCAGTGGTGAACTAACATCA-3' |
| MCM5 | Forward | 5'-GAAGATCCCTGGCATCATCATC-3' |
|  | Reverse | 5'-ATTTGTCGGGCATGATGAAGTA-3' |
| MCM6 | Forward | 5'-TGGCAATGATGAAGTAAAACGG-3' |
|  | Reverse | 5'-ACATTTATGTCCCCTCGAAGAG-3' |
| MCM2 | Forward | 5'-CAGAGCATCTCCATCTCGAAG-3' |
|  | Reverse | 5'-GATGTCAAAGCGTGAGATGATG-3' |
| POLA2 | Forward | 5'-GAGTCGTTCTATGTTTACGCAC-3' |
|  | Reverse | 5'-GACATCCTTCACGAAGTACCTC-3' |
| CARMN exon5 | Forward | 5'-GTTGGAGTCCCGCCACAG-3' |
|  | Reverse | 5'-CATCTCAGACTCCCAACTGACCAG-3' |
| miR143-3p |  | 5’-CCGCTCGATGTCACGAAGTAGAGT-3’ |
| U6 |  | 5’- CGCTTCACGAATTTGCGTGTCAT-3’ |

**Supplementary Table 2: Sequence of siRNAs, miR143-3p inhibitor and miR-143-3p mimics**

| siDROSHA-1 | 5’-GAGUAUUUACUUGCUCAGUACUGAGCAAGUAAAUACUC-3’ |
| --- | --- |
| siDROSHA-2 | 5’-GCUCUGUCCGUAUCGAUCAUGAUCGAUACGGACAGAGC-3’ |
| siDICER1-1 | 5’-GAUCCUAUGUUCAAUCUAAUUAGAUUGAACAUAGGAUC-3’ |
| siDICER1-2 | 5’-CAGCAUACUUUAUCGCCUUAAGGCGAUAAAGUAUGCUG-3’ |
| siMCM5-1 | 5’-GGGUUACCAUCAUGGGCAUAUGCCCAUGAUGGUAACCC-3’ |
| siMCM5-2 | 5’-GCACGGGCUUCACCUUCAAUUGAAGGUGAAGCCCGUGC-3’ |
| miR143-3p inhibitor | 5'-GAGCUACAGUGCUUCAUCUCA-3' |
| hsa-miR143-3p mimics | 5'-UGAGAUGAAGCACUGUAGCUCGAGCUACAGUGCUUCAUCUCA-3’ |

**Supplementary Table 3: miRNAs predicted to bind with MCM5**

| miRNAid | miRNAname |
| --- | --- |
| MIMAT0000101 | hsa-miR-103a-3p |
| MIMAT0000104 | hsa-miR-107 |
| MIMAT0000271 | hsa-miR-214-3p |
| MIMAT0000435 | hsa-miR-143-3p |
| MIMAT0000763 | hsa-miR-338-3p |
| MIMAT0001629 | hsa-miR-329-3p |
| MIMAT0002807 | hsa-miR-491-5p |
| MIMAT0003289 | hsa-miR-620 |
| MIMAT0003320 | hsa-miR-650 |
| MIMAT0004683 | hsa-miR-362-3p |
| MIMAT0004693 | hsa-miR-330-5p |
| MIMAT0004748 | hsa-miR-423-5p |
