# Supplementary material for: LncRNA CARMN overexpression promotes prognosis and chemosensitivity of triple negative breast cancer via acting as miR143-3p host gene and inhibiting DNA replication
Source: J Exp Clin Cancer Res. 2021 Jun 23;40:205. doi: 10.1186/s13046-021-02015-4 (PMC8220716; doi:10.1186/s13046-021-02015-4)
Supplement: Supplementary file 3 — Additional file 3. [file 13046_2021_2015_MOESM3_ESM.docx]

**Supplementary Materials and Methods**

**Patients**

Patients whose tumor tissues were used for evaluating the role of CARMN in predicting response to neoadjuvant chemotherapy all participated in a randomized, open-label, prospective phase III clinical trial of Renji hospital, in which patients received weekly paclitaxel and cisplatin as neoadjuvant chemotherapy for operable local-advanced breast cancer. The neoadjuvant chemotherapy takes 4 cycles (28 days each cycle) before an operation, combined with paclitaxel 80mg/m^2^ on day 1, day 8, day 15, day 22 and cisplatin (cDDP) 25mg/m^2^ on day 1, day 8, day 15. HER2 positive patients can use trastuzumab every week (4mg/kg for the first week and 2mg/kg for the other weeks). Patients with ER or PR positive are randomized given chemotherapy alone or chemotherapy plus endocrine therapy. Response of neoadjuvant chemotherapy was evaluated as primary endpoint in all patients. Details of the clinical trial are also described in previously published article(1).

The response condition of neoadjuvant chemotherapy was distinguished into pathological complete response (pCR) or not depending on pathological results of radical mastectomy after neoadjuvant chemotherapy (NAC) following published article(2).

For determining IHC results, ER≥10% of positive nuclear staining was defined as ER positive. PR≥10% of positive nuclear staining was defined as PR positive. HER2 is detected by both IHC assay and fluorescence in situ hybridization (FISH). A positive FISH result or IHC 3+ was classified to be HER2 positive according to recommendation of American Society of Clinical Oncology/College of American Pathologists (ASCO CAP)(3). IHC assays and FISH results of biopsy tissue were provided by pathology department of Renji Hospital.

**Cell culture**

Breast cancer cell lines and normal breast cell MCF10A were obtained from Renji Hospital, Shanghai Jiao Tong University School of Medicine. MCF10A was cultured with DMEM medium (Gibco, USA) with 5% horse serum (Gibco, USA), hydrocortisone (0.5 lg/ml), insulin (10 lg/ ml), EGF (20 ng/ml), cholera toxin (100 ng/ml) and 1% penicillin-streptomycin. Other cell lines were cultured with DMEM medium (Gibco, USA) with 10% Fetal Bovine Serum (Gibco, USA) and 1% penicillin-streptomycin in 37℃ with 5% CO2.

**RNA isolation and qRT-PCR**

All the tissues were obtained during biopsy and reserved in liquid nitrogen immediately. Total RNA of tissues or cells was extracted by TRIzol (Invitrogen, USA). Isolated RNA was reverse-transcribed into cDNA with Reverse Transcription Kit (Takara, Japan). Reverse-transcription of miRNA was done with miRcute plus miR first-strand cDNA synthesis kit (Tiangen, China). QPCR was done with SYBR Premix Ex TaqII (Takara, Japan) and miRcute plus miR qPCR detection kit (Tiangen, China). RT-qPCR result was detected with light cycler instrument (LightCycler 480 II, Roche, Mannheim, Germany) under following procedure: for lncRNA and protein coding RNA, 95℃ 5min, 40 cycles of 95℃ 10s and 60℃ 1min; for miRNA, 95℃ 15min, 40 cycles of 94℃ 20s and 60℃ 34s. The expression of lncRNA and protein coding RNA was normalized by β-actin and miRNA expression was normalized by U6. The primers of detected RNAs are listed in **Supplementary Table 1**. Expression of detected RNAs was calculated by 2^-ΔΔCT^, and each sample has 3 replications.

**Cell transfection with lentivirus, miRNA inhibitor and plasmid**

To stably overexpress CARMN, MDA-MB-231, MDA-MB-468 and MCF10A cells were transfected with lentivirus vectors overexpressing CARMN or control ones purchased from GenePharma (Shanghai, China). Cells were seeded in 6-well plates till covering 50% of the plate, the cells were treated with the lentivirus under multiplicity of infection (MOI) of 5 and polybrene (5μg/mL). After 2 days, the cells were cultured in culture medium adding Puromycin (2μg/mL, Sigma, USA) to screen successfully transfected cells. The cells remained were collected for further use.

CARMN smart silencer(8) was bought from Ribobio (Guangzhou, China). MiR143-3p inhibitor and inhibitor control (GenePharma, Shanghai, China), miR143-3p mimics (purchased from Genomeditech, China), siRNA of DROSHA/DICER1/MCM5 (purchased from Genomeditech, China), plasmid overexpressing exon5 of CARMN (purchased from Genechem, Shanghai, China), whose sequence are all listed in **Supplementary Table 2.** Cells were cultured in 6-well plate in density of 2×10^5^ cells/well and were transfected assisted with Polyplus Transfection jetPRIME (France). After 48h, the cells were collected for detecting efficiency and further use.

**Cell proliferation and migration assay**

Cells were seeded in 96-well plate with 2×10^3^ cells/well. Once cells were adhered in the plate, cell proliferation was detected at 0h, 24h, 48h and 72h with Cell Counting Kit-8 (CCK-8, Dojindo, Japan) and detected with 450 nm absorbance by EPOCH2 microplate reader (Biotek, USA). Wells with culture medium without cells were set as blank.

For colony formation assay, Cells were seeded in 6-well plate with 2×10^3^ cells/well, incubated in 37℃ and 5% CO2. After 14 days, the plates were observed under microscope, fixed with 4% paraformaldehyde for 30 min and stained with crystal violet for 30 min under room temperature.

For migration assay, Cells were seeded in 24-well transwell plate upper chamber with 4×10^5^ cells/well. Cells were cultured in serum-free medium in the upper chamber and medium with serum in the lower chamber, under 37℃ and 5% CO2. After 24h, the cells at bottom of the filters were fixed and stained for obtaining and counting.

**Drug sensitivity assay**

The cells were seeded in 96-well plate with 1×10^4^ cells/well. Cells were treated with different concentration of cisplatin (CSNpharm, Chicago, USA): 100, 50, 25, 12.5, 6.25, 3.13, 1.56, 0.78, 0.39, 0.20, 0.10 and 0 μg/ml). Cell activity was detected by CCK8 assay after 24h.

**EdU cell proliferation assay**

EdU staining was used for evaluating DNA replication activity and cell proliferation, with EdU Cell Proliferation Kit with Alexa Fluor 488 (BeyoClick, China). Cells were seeded in 24-well plate with 5×10^4^ cells/well. Cells were treated with 5μg/ml cisplatin or blank for 24h. Cells were stained with EdU following manufacture’s protocol Hoechst was stained as well for identifying cell nucleus.

**Western blot and antibodies**

For isolating protein, different cells were lysed with RIPA Buffer and protease inhibitor. Isolated protein was qualified with BCA methods. Equal amount of protein was loaded in SDS/PAGE gels and electrophoresed under 60 mV for 30min and then 120 mV for 30min. Then SDS/PAGE gels were transferred to nitrocellulose membranes under 100 mV for 60 min, and the membranes were blocked with blocking buffer (5% BSA) under room temperature for 1h. After blocking, the membranes were incubated in primary antibodies at 4℃ overnight. After washing the membranes with TBST 3 times for 20 mins, the membranes were incubated in secondary antibodies under room temperature for 1h. After washing the membranes with TBST 3 times for 20 mins, enhanced chemiluminescence (ECL) was performed with Immobilon Western Chemiluminescent HRP Substrate (Millipore, Billerica, USA) and Chemidoc Touching Imaging System (Biorad, California, USA) was used for observing results. Primary antibodies of MCM5 (11703-1-AP, anti-rabbit 1:500) and ACTIN (ab49900, HRP, 1:1000) were purchased from Proteintech (Chicago, USA) and Abcam (Cambridge, UK) respectively. Secondary antibody (A0208, 1:2000) was bought from Beyotime (Shanghai, China). Quantification of results was implied with software ImageJ (v1.52, NIH, USA).

**Immunofluorescence**

Immunofluorescence was performed for detecting DAPI and β-tubulin. Cells were seeded in 6-well plate with 2×10^5^ cells/well. The cells were fixed with 4% paraformaldehyde for 15 min under room temperature and permeabilized with 0.05% Triton X-100 for 1 min. Then 1% BSA was used for blocking cells for 1h. Later, the cells were stained with DAPI stain and β-tubulin antibody (Servicebio, Hubei, Wuhan). Images of immunofluorescence were obtained with confocal-scope (Carl Zeiss). Software Caseviewer (V2.0) was used for dealing photos.

**Cell cycle and apoptosis assay**

Flow cytometry was used for cell cycle and apoptosis assay. For cell cycle assay, cells were collected and stained with propidium iodide (PI) using Cell Cycle Assay Kit - PI/RNase Staining (Dojindo, Japan) following manufacture’s protocol. For cell apoptosis assay, cells were seeded in 6-well plate in density of 5×10^5^cells/well. Seeded cells were treated with cDDP (5μg/ml) or blank control for 24h. Later, cells were collected and labeled with Annexin-V FITC and PI using Apoptosis Detection Kit (Dojindo, Japan). LSRFortessa (BD) was then used for flow cytometry analysis. Software Flowjo (v10.0.7r2) and Modfit (v4.0) was applied for data analysis.

**RNA Transcriptome sequencing**

RNA-Seq was performed in CARMN overexpressed and control MDA-MB-231 cells. For library preparation, 1 μg RNA from each sample was separated for RNA sample preparations. Sequencing libraries generation was performed using NEBNextR UltraTM Directional RNA Library Prep Kit for IlluminaR (NEB, USA) following manufacturer’s protocol. Then, the clustering of the samples was carried out with cBot Cluster Generation System using TruSeq PE Cluster Kit v4-cBot-HS (Illumina) according to the manufacturer’s protocol. Illumina Hiseq Xten platform was used for library preparations sequence. For comparative analysis, the adaptor sequences and low-quality sequence reads were first removed from the data sets, and raw data were transformed into clean reads. Gene expression levels were calculated by fragments per kilobase of transcript per million fragments mapped (FPKM). Differential expression analysis of two different CARMN expression condition groups was performed with R package “DESeq” under threshold of adjusted P-value < 0.05 and absolute value of fold change>1.5.

**Luciferase Reporter Assay**

First, binding site of miR143-3p and MCM5 was predicted with STARBASE(4) (http://starbase.sysu.edu.cn/index.php). For dual-luciferase reporter assays, luciferase reporter vectors (pMIR-CMV Vector, Promega, Madison, WI, USA) with wild-type MCM5 or mutant MCM5 in predicted binding site were constructed. MDA-MB-231 cells were co-transfected with luciferase reporter vectors and miR143-3p or negative control mimics. Reporter luciferase activity was detected after 48h by Dual-Luciferase Reporter Assay System (Promega) with Renilla luciferase activity for normalization.

**Experiments in vivo**

Female BALB/c nude mice (SLAC, Shanghai, China) were used for breast cancer xenograft model. CARMN overexpressed or control MDA-MB-468 cells was suspended with 100μL PBS in density of 1×10^7^ and then injected into subcutaneous flanks of 5-week-old nude mice. The volume of the tumors was measured every week. Tumor volume was calculated as length × width ^2^ × 0.5. After tumors reach 100 mm^3^, mice were randomly separated into 4 groups: Control + saline, Control + cDDP, CARMN + saline, CARMN + cDDP. Saline or cDDP was injected intraperitoneally every 3 days, with cDDP concentration of 5 mg/kg. All mice were sacrificed 6 weeks after injection and xenografts were taken out for weighting. Experiments in vivo were performed following protocol approved by Shanghai Jiao Tong University Institutional Animal Care and Use Committee and Renji Hospital Animal Care guidelines. To minimize animal suffering, all efforts were made.

**Immunohistochemistry and terminal deoxynucleotidyl transferase (TdT) dUTP nick-end labeling (TUNEL) assay**

IHC assay of Ki67, PCNA were performed on paraffin-embedded xenografts. Primary antibody of Ki67 (anti-rabbit 1:500) and PCNA (anti-mouse, 1:2000) were purchased form Sevicebio (Wuhan, Hubei, China). After conjugated with anti-rabbit or anti-mouse HRP conjugated antibodies, DAB reaction was performed for visualization. Tumor cells with more than 1% of positive staining were defined as Ki67 or PCNA positive. TUNEL assay of xenografts was implied with TUNEL kit from Roche (Basel, Switzerland) in xenografts following manufacture’s protocol. Images of TUNEL were obtained with confocal-scope (Carl Zeiss). Software Caseviewer (V2.0) was used for dealing photos.

**Bioinformatic analysis**

The differentially expressed lncRNA between breast cancer tissue and normal tissue in TCGA dataset was analyzed by R package “TCGAbiolinks”. GEO dataset GSE45827, GSE31192, GSE3744and GSE21422 was also used for screening differentially expressed lncRNA, with R package “limma”. Expression of CARMN in breast tumor and normal tissue in TCGA set was analyzed by online tool GEPIA (http://gepia.cancer-pku.cn/index.html)^9^. The survival plot in breast cancer cohort were analyzed with K-M plotter (http://kmplot.com/analysis/index.php?p=background)^10^. KEGG and GO pathway enrichment was carried out with R package “clusterProfiler”, “DOSE” and “enrichplot” ^11, 12^. GSEA pathway enrichment was carried out with software GSEA (version 4.0.1)^13^. Genome information of CARMN was obtained from ENSEMBL (http://uswest.ensembl.org) and NCBI gene (https://www.ncbi.nlm.nih.gov). MiRNA sequence was obtained from miRbase (http://www.mirbase.org).

**Statistical analysis**

Statistical analysis and figures were conducted with R (3.4.3) and Graphpad Prism (8.1.1). LASSO regression conducted by 10-fold cross validation following minimum criteria^14^ was used for selecting parameters related with NAC pathological complete response in multivariate logistics model, with R package “glmnet”. Factors participated in lasso regression include CARMN expression, ER, PR, HER2, Ki67, menstruation status, age, T stage, N stage, body mass index (BMI) and usage of gonadotropin-releasing hormone agonist (GNRHa). Parameters are included in the logistics model when λ in reaches minimum. Nomogram was carried out with R package “rms”. ROC curve was performed by R package “pROC”. Calibration curve of the prediction model was done by R package “foreign”. Decision curve is realized by R package “rmda”. Cox model of DFS was tested by log-rank test whose survival plot is done by R package “survminer”. The expression of CARMN in normal or tumor tissue is tested by Student t test, and bar plot of CARMN expression is done with R package “ggplot2”. Baseline of training set and validation is compared with Chi square test. The expression of CARMN in different subtypes is compared by one-way anova. Two-side p value <0.05 was considered as statistically significant.

**References**

1. Zhou L, Xu S, Yin W, Lin Y, Du Y, Jiang Y*, et al.* Weekly paclitaxel and cisplatin as neoadjuvant chemotherapy with locally advanced breast cancer: a prospective, single arm, phase II study. *Oncotarget* 2017;**8**(45):79305-14 doi 10.18632/oncotarget.17954.

2. Rodenhuis S, Mandjes IAM, Wesseling J, van de Vijver MJ, Peeters M, Sonke GS*, et al.* A simple system for grading the response of breast cancer to neoadjuvant chemotherapy. *Ann Oncol* 2010;**21**(3):481-7 doi 10.1093/annonc/mdp348.

3. Wolff AC, Hammond MEH, Allison KH, Harvey BE, Mangu PB, Bartlett JMS*, et al.* Human Epidermal Growth Factor Receptor 2 Testing in Breast Cancer: American Society of Clinical Oncology/College of American Pathologists Clinical Practice Guideline Focused Update. *J Clin Oncol* 2018;**36**(20):2105-22 doi 10.1200/JCO.2018.77.8738.

4. Li JH, Liu S, Zhou H, Qu LH, Yang JH. starBase v2.0: decoding miRNA-ceRNA, miRNA-ncRNA and protein-RNA interaction networks from large-scale CLIP-Seq data. *Nucleic Acids Res* 2014;**42**(Database issue):D92-7 doi 10.1093/nar/gkt1248.
